# Smoother Alchemical Transformations via Enveloping Distribution Sampling for Free-Energy Estimation

**DOI:** 10.1021/acs.jctc.6c00581

**Published:** 2026-07-13

**Authors:** Shu-Yu Chen, Enrico Ruijsenaars, Philippe H. Hünenberger, Sereina Riniker

**Affiliations:** Department of Chemistry and Applied Biosciences, ETH Zurich, Vladimir-Prelog-Weg 2, Zurich 8093, Switzerland

## Abstract

The accuracy of the computational estimation of relative free energies (e.g., for solvation or protein–ligand binding) depends on the smoothness of the phase-space transformation between the two alchemical end-states. A smooth transformation ensures sufficient phase-space overlap between the neighboring intermediate states connecting the two end-states in equilibrium (EQ) simulations and generates less dissipative work in nonequilibrium (NEQ) simulations. The conventional energy interpolation (EI) coupling scheme constructs the intermediate states by linearly combining the end-state potentials. We show that the enveloping distribution sampling (EDS) coupling scheme, a generalization of EI where the corresponding Boltzmann factors are linearly combined, represents a much more flexible alternative. Through the use of a negative smoothing parameter, the EDS scheme increases the local curvature of the sampling phase space along the transformation axis, thereby avoiding phase transitions and creating a smoother transformation. We validate this behavior in increasingly complex settings, from harmonic oscillators and Ising model systems to absolute hydration free-energy (AHFE) calculations on the FreeSolv data set. EDS consistently yields more accurate and statistically robust free-energy estimates compared to the conventional EI scheme for the model system calculations, while a clear advantage is observed for AHFE in the NEQ regime, where less dissipative transitions lead to more reliable free-energy estimates.

## Graphical abstract

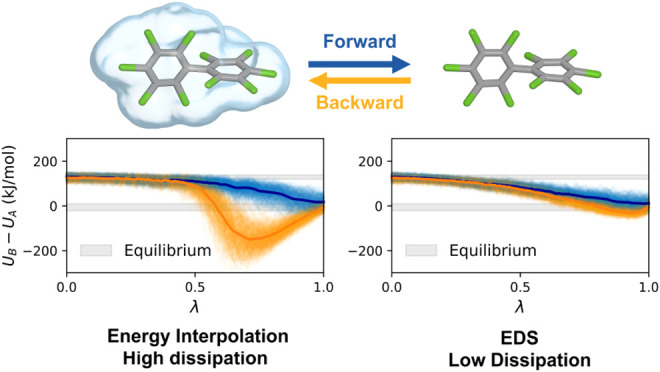

## Introduction

Free energy is a fundamental equilibrium property of thermodynamic systems and is a quantity of high relevance in biology,^1–5^ materials science,^6–8^ and drug discovery.^9,10^ If the equilibrium distributions (ensembles) of two systems overlap sufficiently, the free-energy difference can be obtained using free-energy perturbation (FEP)^11^ or the Bennett acceptance ratio (BAR)^12^ methods. However, in most real-world applications, the phase-space overlap between the ensembles of interest is insufficient. Thus, to connect the two ensembles, one can construct nonphysical intermediate states along an alchemical coordinate through the coupling parameter λ ∈ [0,1]. The process of sampling the intermediate states is known as an alchemical transformation.^9,11,13,14^

Alchemical transformations can be carried out using equilibrium (EQ) and nonequilibrium (NEQ) methods. Conventional EQ methods such as FEP, thermodynamic integration (TI),^13–16^ and multistate BAR (MBAR)^17^ require the selection of discrete intermediate states, either *a priori* or adaptively on-the-fly. The quality of the free-energy estimates then depends on the convergence in each λ-state and on the phase-space overlap between neighboring states. As an alternative to independently sampling the discrete λ-states, other EQ schemes such as enveloping distribution sampling (EDS)^18,19^ and λ-dynamics^20–22^ were designed to sample multiple ensembles in one simulation. Despite promising gains in sampling efficiency, the unmodified use of these methods suffers from severe convergence issues for more complex systems due to energy barriers, and the sampling must generally be enhanced (e.g., RE-EDS^23,24^ using Hamiltonian replica exchange^25^). Convergence issues are also frequently affecting conventional EQ alchemical-transformation schemes, such as order/disorder transitions^26^ and end-point catastrophes,^15,27^ which need to be mitigated using soft-core potentials^27–30^ or alchemical perturbation functions.^26^ In contrast, NEQ methods based on Jarzynski’s equality (JE)^31^ and the Crooks fluctuation theorem (CFT)^32,33^ estimate the free-energy difference by switching the Hamiltonian from one state to the other along the alchemical coordinate via a time-dependent schedule λ­(*t*). Because they continuously drive the system along λ, NEQ methods can traverse energy barriers by forcing the passage through transition regions, reducing the trapping in local minima. However, abrupt changes in phase space during the λ-switching process can generate large dissipative work (*W*_*d*_), which reduces the quality of the free-energy estimation.^34–37^ Although NEQ methods have recently been applied to the estimation of binding free energies of small molecules and host–guest systems^38–41^ and buried water molecules in proteins,^42^ there is currently no established consensus concerning which of the established EQ methods or the recently emerging NEQ methods are more efficient.^37,43–45^

In the present study, we investigate the fundamental properties of the EDS coupling scheme and show that with proper parameter choices, the transformation process constructed by this scheme is in most cases smoother than the conventional energy interpolation (EI) coupling scheme. We compare the performance of the two coupling schemes in different systems with increasing complexity: 1D harmonic oscillators, 2D Ising models, and absolute hydration free-energy calculations of molecules from the FreeSolv^46^ data set. We show that the EDS coupling scheme consistently provides equal or improved performance over the EI alternative for both EQ and NEQ sampling methods. The EDS coupling scheme, as a generalization of the EI scheme with an additional parameter *s*, offers more control over the nonequilibrium protocol and thus an opportunity to perform the transformation with a thermodynamic length^47,48^ lower than that which could be reached using the EI coupling scheme.

## Theory

### EQ and NEQ Methods in Free-Energy Calculations

Consider a thermodynamic state *A* characterized by a potential-energy function *U*_*A*_(**r**), where $r\in R^{d}$ is a given configuration in *d* dimensions. The corresponding Helmholtz free energy *F*_*A*_ in the canonical ensemble is given by

1 $$F_{A}=-\beta^{-1}\ln\int dre^{-\beta U_{A}\left(r\right)}$$

where β = 1/(*k*_B_ *T*) is the reciprocal temperature with the Boltzmann constant *k*_B_ and absolute temperature *T*. If *n*_*A*_ samples are collected from ensemble *A*, its free-energy difference to another thermodynamic state *B* described by the potential-energy function *U*_*B*_(**r**) can be estimated by Zwanzig reweighting,^11^ also known as FEP

2 $$\Delta F_{\mathrm{AB}}^{FEP}=F_{B}-F_{A}=-\beta^{-1}\ln\left(\frac{1}{n_{A}}\sum_{i}^{n_{A}}e^{-\beta \Delta U_{i}}\right)$$

where Δ*U*_*i*_ = Δ*U*(**r**_**i**_) = *U*_*B*_(**r**_**i**_) – *U*_*A*_(**r**_**i**_). If both ensembles *A* and *B* are sampled at equilibrium with *n*_*A*_ and *n*_*B*_ uncorrelated samples, respectively, the free-energy difference can be estimated by solving the BAR equation^12^

3 $$\sum_{i}^{n_{A}}\frac{1}{1+\frac{n_{A}}{n_{B}}e^{\beta \left(\Delta U_{i}-\Delta F_{\mathrm{AB}}^{\mathrm{BAR}}\right)}}=\sum_{j}^{n_{B}}\frac{1}{1+\frac{n_{B}}{n_{A}}e^{-\beta \left(\Delta U_{j}-\Delta F_{\mathrm{AB}}^{\mathrm{BAR}}\right)}}$$

The uncertainty of the estimated free-energy differences will be large if the two end-state ensembles do not have sufficient overlap in the Δ*U* distribution.^12^ To overcome this problem, intermediate states, usually controlled by a discrete set of coupling parameter values λ_*k*_ ∈ [0,1], are needed to bridge the two end states. If sufficient overlap in the Δ*U* distributions is created between all neighboring intermediate ensembles, more reliable free-energy differences can be obtained by estimators such as TI^13,49^ or MBAR.^17^

In contrast to EQ methods, NEQ approaches connect the two end-states by switching the parameter λ­(*t*) as a function of time *t* between zero and one in a continuous fashion and measuring the work *W* performed on the system by the corresponding λ-force. By drawing *n*_*f*_ samples from ensemble *A* and recording the work associated with each forward transformation process (*A* → *B*), the free-energy difference can be obtained by JE^31,50^

4 $$\Delta F_{\mathrm{AB}}^{\mathrm{JE}}=-\beta^{-1}\ln\left(\frac{1}{n_{f}}\sum_{i}^{n_{f}}e^{-\beta W_{i}}\right)$$

If the NEQ process is performed in both directions using *n*_*f*_ and *n*_*b*_ forward and backward shooting events, respectively, CFT^32,33^ can be used to estimate the free-energy difference, usually in a BAR-like form (CFT-BAR)^36^

5 $$\sum_{i}^{n_{f}}\frac{1}{1+\frac{n_{f}}{n_{b}}e^{\beta \left(W_{i}-\Delta F_{\mathrm{AB}}^{\mathrm{CFT}}\right)}}=\sum_{j}^{n_{b}}\frac{1}{1+\frac{n_{b}}{n_{f}}e^{\beta \left(W_{j}+\Delta F_{\mathrm{AB}}^{\mathrm{CFT}}\right)}}$$

Similarly to the BAR equation in the EQ approaches, the accuracy of CFT-BAR relies on the overlap between the forward and negative backward work distributions. Note that eqs 2 and 3 can be viewed as a limiting case of eqs 4 and 5, respectively, where the transformations are performed in a single step.

The overlap between the forward and backward work distributions relies on the *reversibility* of the NEQ protocol. In fast transformations between two poorly overlapping ensembles, the phase-space overlap between the ensembles at time *t* and *t* + *dt* is small for any given path, and the dynamics of the system cannot catch up with the change of the underlying ensemble. As a result, the system is driven far from equilibrium, leading to large dissipative work and broad, weakly overlapping work distributions in the forward and backward directions. This poor overlap significantly increases the statistical uncertainty of free-energy estimators based on fluctuation relations such as JE or CFT.^51^ This dependence of the NEQ estimator efficiency on dissipative work and work-distribution overlap has been emphasized in previous methodological studies of NEQ free-energy calculations, where reducing dissipation is a central route to improving convergence.^43,52,53^ In the following, we use the term *smooth* to describe an alchemical transformation that avoids abrupt changes in the equilibrium ensembles sampled along the protocol. In NEQ calculations, this corresponds to a more reversible switching process at a given switching time. The degree of irreversibility is quantified by the dissipative works, ⟨*W*_*d*_ ⟩_forward_ = ⟨*W*⟩_forward_ – Δ*F*_*AB*_ for the forward process and ⟨*W*_*d*_⟩_backward_ = ⟨*W*⟩_backward_ + Δ*F*_*AB*_ for the backward process, which vanish in the reversible limit and increase as the system is driven further from equilibrium. Thus, a smoother NEQ transformation produces less dissipative work, better overlap between forward and backward work distributions, and more statistically efficient free-energy estimates.

### EI and EDS Coupling Schemes

To generate the potentials of intermediate states that interpolate between the two end-state potentials *U*_*A*_(**r**) and *U*_*B*_(**r**) as a function of the coupling parameter λ, an appropriate coupling scheme must be defined. A popular coupling scheme is EI,

6 $$U_{\mathrm{EI}}\left(r,\lambda \right)=\left(1-\lambda \right)U_{A}\left(r\right)+\lambda U_{B}\left(r\right)$$

in which the force $\left(f_{\mathrm{EI}}\in R^{d}\right)$ and the Hessian matrix $\left(H_{\mathrm{EI}}\in R^{d\times d}\right)$ are also linearly interpolated

7 $$f_{\mathrm{EI}}\left(r,\lambda \right)=\left(1-\lambda \right)f_{A}\left(r\right)+\lambda f_{B}\left(r\right)=f_{A}\left(r\right)+\lambda \left(f_{B}\left(r\right)-f_{A}\left(r\right)\right)$$

and

8 $$H_{\mathrm{EI}}\left(r,\lambda \right)=\left(1-\lambda \right)H_{A}\left(r\right)+\lambda H_{B}\left(r\right)$$

The last equality in eq 7 shows that the transformation process can be seen as a perturbation in state *A* by a perturbing force λ­(**f**_*B*_(**r**) – **f**_*A*_(**r**)). In contrast, the EDS coupling scheme builds intermediate potentials by linearly interpolating the Boltzmann factors at a scaled reciprocal temperature *s*β

9 $$U_{\mathrm{EDS}}\left(r,\lambda ;s,E\left(\lambda \right)\right)=-{\left(s\beta \right)}^{-1}\ln\left[\left(1-\lambda \right)e^{-s\beta U_{A}\left(r\right)}+\lambda e^{-s\beta \left(U_{B}\left(r\right)-E\left(\lambda \right)\right)}\right]$$

where *s* is the smoothing parameter and *E*(λ) is the λ-dependent energy offset. Here, we adopted the form proposed by König et al.^54,55^ Since the parameter *s* is a constant in the present study and *E*(λ) is a function of λ, we do not show them explicitly in the following equations for simplicity. At λ = 0 and λ = 1, *U*_EDS_ recovers the physical end-point potentials, up to an offset

10 $$U_{\mathrm{EDS}}\left(r,0\right)=U_{A}\left(r\right)$$

11 $$U_{\mathrm{EDS}}\left(r,1\right)=U_{B}\left(r\right)-E\left(1\right)$$

The force resulting from the EDS coupling scheme is a linear combination of end-state forces with an effective coupling parameter λ_EDS_(**r**, λ) ∈ [0,1] that depends not only on λ, but also on the instantaneous configuration **r**

12 $$f_{\mathrm{EDS}}\left(r,\lambda \right)=\left(1-\lambda_{\mathrm{EDS}}\right)f_{A}\left(r\right)+\lambda_{\mathrm{EDS}}f_{B}\left(r\right)=f_{A}\left(r\right)+\lambda_{\mathrm{EDS}}\left(f_{B}\left(r\right)-f_{A}\left(r\right)\right)$$

where

13 $$\lambda_{\mathrm{EDS}}\left(r,\lambda \right)=\lambda_{\mathrm{EDS}}\left(r,\lambda ;s,E\left(\lambda \right)\right)=\frac{\lambda }{\lambda +\left(1-\lambda \right)e^{s\beta \left(\Delta U\left(r\right)-E\left(\lambda \right)\right)}}$$

The effective coupling parameter λ_EDS_ has a sigmoidal shape as a function of *s*β­(Δ*U*(**r**) – *E*(λ)) and equals λ when *sβ*(Δ*U*(**r**) – *E*(λ)) = 0 (Figure 1A). In the limit of vanishing *s*, the EDS potential becomes equivalent to the EI potential, up to a configuration-independent constant

14 $$\lim_{s\to 0}U_{\mathrm{EDS}}\left(r,\lambda \right)=U_{\mathrm{EI}}\left(r,\lambda \right)-\lambda E\left(\lambda \right)$$

with a finite first derivative with respect to *s*

15 $$\lim_{s\to 0}\frac{\partial U_{\mathrm{EDS}}}{\partial s}=-\frac{\beta }{2}\lambda \left(1-\lambda \right){\left[U_{A}\left(r\right)-U_{B}\left(r\right)+E\left(\lambda \right)\right]}^{2}$$

**Figure 1:**
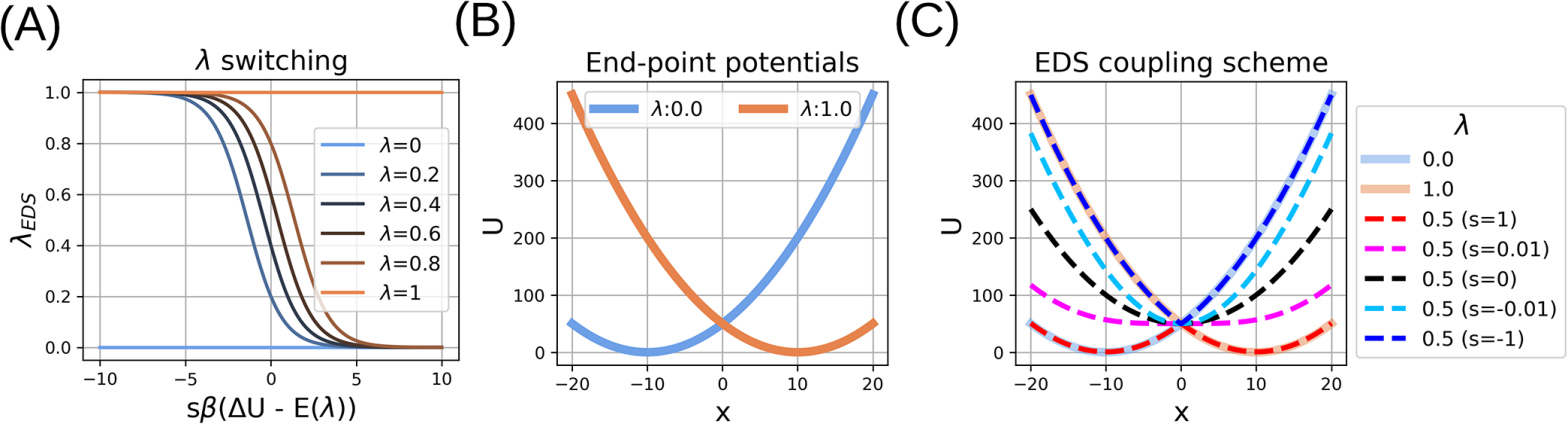
Illustration of the EDS coupling scheme and the effects of the parameters *s* and *E*(λ). (A): λ_EDS_ as a function of *s*β­[Δ*U*(**r**) – *E*(λ)] from eq 13 at different λ-values. (B): Two end-state potentials *U*_*A*_(*x*) = 0.5 (*x* + 10)^2^ and *U*_*B*_(*x*) = 0.5 (*x* – 10)^2^ of system HO_1_. (C): Intermediate states constructed by the EDS coupling scheme at λ = 0.5 using *E*(λ) = 0 along with different *s*-values (dashed lines). The end-state potentials are reconstructed at λ = 0 and λ = 1 (light blue and light orange solid lines).

As a simple example, Figure 1B considers two 1D harmonic oscillators with the same force constant and shifted equilibrium positions as the two end-states. The intermediate states using the EDS coupling scheme at λ = 0.5 with *E*(λ) = 0 and different *s*-values are shown in Figure 1C. In previous work,^18,23,54–56^ only positive *s*-values were considered, in which case the energy landscape is biased toward the end-state potential with a lower energy in a given configuration *x* (Figure 1C). Compared to the EI coupling scheme (*s* = 0), the EDS intermediate states constructed with *s* > 0 cover a broader phase space. This permits the calculation of the free-energy difference between the two end-states from a single ensemble using Zwanzig reweighting (eq 2). In contrast, the use of a negative *s*-value biases the energy landscape toward the end-state potential with the highest energy in a given configuration *x*, and leads to a narrower phase-space overlap with the end-states compared to the EI scheme (*s* = 0). In the following, we discuss how the choices of the *s*-parameter and of the *E*(λ) function in the EDS coupling scheme affect the constructed intermediate states.

#### Modulation of the Local Curvature

To show that the effects observed in Figure 1 also apply to systems beyond harmonic oscillators, we derived an analytical expression for the local curvature. The Hessian matrix of the intermediate state in an EDS coupling scheme reads

16 $$H_{\mathrm{EDS}}=\left(1-\lambda_{\mathrm{EDS}}\right)H_{A}+\lambda_{\mathrm{EDS}}H_{B}-s\beta \lambda_{\mathrm{EDS}}\left(1-\lambda_{\mathrm{EDS}}\right)\left(f_{B}-f_{A}\right){\left(f_{B}-f_{A}\right)}^{T}$$

eq 16 can again be viewed as a perturbation of the EI Hessian (the first two terms are the same as in eq 8 aside from λ) along the perturbation axis of $v=\frac{f_{B}-f_{A}}{∥f_{B}-f_{A}∥}$ . Since the matrix λ_EDS_(1 – λ_EDS_)­(**f**_*B*_(**r**) – **f**_*A*_(**r**)) (**f**_*B*_(**r**) – **f**_*A*_(**r**))^*T*^ is positive-definite, a positive *s*-parameter can decrease the local curvature along the **v** axis whereas a negative *s* can increase it. For a given configuration **r** and coupling parameter λ, there exists a critical value

17 $$s^{*}\left(r,\lambda \right)=\frac{v^{T}H_{\mathrm{EI}}{|}_{\lambda =\lambda_{\mathrm{EDS}}}v}{\beta \lambda_{\mathrm{EDS}}\left(1-\lambda_{\mathrm{EDS}}\right)∥f_{B}-f_{A}{∥}^{2}}$$

with which the curvature along the **v**-axis becomes zero. Since the transformation is performed along the **v**-axis, using an *s* < s* avoids an unstable intermediate state with local negative curvature. In the simple example of a 1D harmonic oscillator (HO_1_) (Figure 1B), the corresponding s* is equal to $\frac{4}{\beta }{\left[k{\left(x_{B}-x_{A}\right)}^{2}\right]}^{-1}=0.01$ at *x* = 0 and λ = 0.5, where the curvature reaches zero. Notably, the difference between **H**_EDS_ and **H**_EI_ also implies that an EDS coupling scheme in general does not follow the same local minimum-energy path (MEP) along the λ-coordinate as the EI coupling scheme. More details on how the MEP is affected by the EDS coupling scheme are provided in Section S1 in the Supporting Information.

#### Modulation of the Δ*U* Distribution

While the expression for the Hessian matrix in eq 16 highlights how curvature is modulated for individual configurations, we are also interested in how the EDS coupling scheme modulates the phase-space distribution along the transformation process. At equilibrium, intermediate ensembles from the EI and EDS coupling schemes follow the Boltzmann distributions

18 $$p_{\mathrm{EI}}\left(r,\lambda \right)=\frac{e^{-\beta U_{\mathrm{EI}}\left(r,\lambda \right)}}{Z_{\mathrm{EI}}\left(\lambda \right)}$$

and

19 $$p_{\mathrm{EDS}}\left(r,\lambda \right)=\frac{e^{-\beta U_{\mathrm{EDS}}\left(r,\lambda \right)}}{Z_{\mathrm{EDS}}\left(\lambda \right)}$$

where *Z*_EI_ and *Z*_EDS_ are the partition functions using the two coupling schemes. Using a Dirac delta function δ (·), the distributions can be mapped to the Δ*U* domain

20 $${\mathrm{p̃}}_{\mathrm{EI}}\left(\Delta U,\lambda \right)=\int \mathrm{dr}\delta \left(\Delta U-\left[U_{B}\left(r\right)-U_{A}\left(r\right)\right]\right)p_{\mathrm{EI}}\left(r,\lambda \right)$$

21 $${\mathrm{p̃}}_{\mathrm{EDS}}\left(\Delta U,\lambda \right)=\int \mathrm{dr}\delta \left(\Delta U-\left[U_{B}\left(r\right)-U_{A}\left(r\right)\right]\right)p_{\mathrm{EDS}}\left(r,\lambda \right)$$

From eqs 6 and 9, the potential-energy difference between the intermediate states at λ constructed by the EDS and EI coupling schemes is an explicit function of Δ*U*(**r**) – *E*(λ),

22 UEI→EDS(r,λ)=UEDS(r,λ)−UEI(r,λ)=−λ[ΔU(r)−E(λ)]−(sβ)−1⁡ln[(1−λ)+λe−s[ΔU(r)−E(λ)]]

This permits an analytical expression of the relative equilibrium probability density between the EDS and EI coupling schemes in the Δ*U* domain as a function of λ

23 $${\mathrm{p̃}}_{\mathrm{EDS}}\left(\Delta U,\lambda \right)=\frac{Z_{\mathrm{EI}}\left(\lambda \right)}{Z_{\mathrm{EDS}}\left(\lambda \right)}\phi \left(\Delta U\left(r\right)-E\left(\lambda \right),\lambda \right){\mathrm{p̃}}_{\mathrm{EI}}\left(\Delta U,\lambda \right)$$

where

24 $$\phi \left(\Delta U\left(r\right)-E\left(\lambda \right),\lambda \right)=\exp\left(-\beta U_{\mathrm{EI}\to \mathrm{EDS}}\left(r,\lambda \right)\right)$$

The first factor on the right-hand side of eq 23 is a λ-dependent but configuration-independent normalization factor, while the second factor ϕ is a λ-dependent probability scaling factor in the Δ*U* domain. When *s* < 0 and for any λ ∈ (0,1), ϕ is a concave function of Δ*U*(**r**) – *E*(λ) with a maximum at Δ*U*(**r**) = *E*(λ) (Figure 2). In other words, the distribution in the Δ*U* domain is biased toward the selected energy offset *E*. When using EQ methods like FEP (eq 2) and BAR (eq 3), the error often depends primarily on the overlap of the Δ*U* distributions, rather than the *d*-dimensional configurations. In this case, the scaling function ϕ with *s* < 0 provides a practical tool to reshape the Δ*U* distributions and modulate the overlap between neighboring λ-windows by an appropriate choice of *E*(λ). For this reason, the remainder of this work is devoted to further investigation of how alchemical transformations can be guided by modulating the Δ*U* distribution with EDS intermediate states using negative *s*-values.

**Figure 2:**
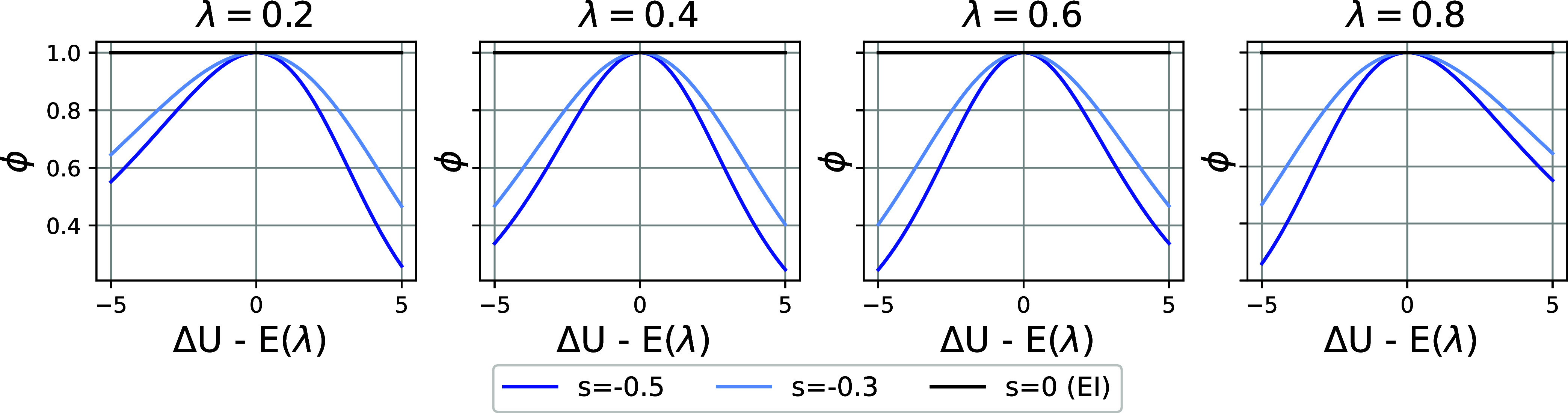
Probability density scaling factor ϕ in the Δ*U* domain from eq 24 as a function of Δ*U* – *E* at λ = {0.2, 0.4, 0.6, 0.8}.

### Selection of *E*(λ) and *s*

To enable a smooth transformation between the end-states, a naive selection of *E*(λ) could be a linear interpolation *E*_lin_ between the means of the two Δ*U* distributions from the end-state ensembles,

25 $$E_{\mathrm{lin}}\left(\lambda \right)=\left(1-\lambda \right)\langle \Delta U\rangle_{0}+\lambda \langle \Delta U\rangle_{1}$$

where the angular brackets denote an ensemble average at the indicated λ. To make sure that the Hamiltonian defined by the EDS coupling scheme interpolates between the end-state forces (i.e., 0 < λ_EDS_ < 1), the inverse of the parameter *s* and the expected range of β­(Δ*U*(**r**) – *E*(λ)) must be of comparable magnitudes, so that the sigmoid-like function of λ_EDS_ (eq 13 and Figure 1A) operates in the linear regime. Using information from the end-state ensembles, we propose a selection of *s* that also involves the corresponding standard deviations σ_0_^Δ*U*^ and σ_1_^Δ*U*^ as well as a unitless scaling parameter α for further empirical tuning

26 $$s_{\alpha }=\frac{\alpha }{\beta }{\left[\left|\langle \Delta U\rangle_{0}-\langle \Delta U\rangle_{1}\right|+2\sigma_{0}^{\Delta U}+2\sigma_{1}^{\Delta U}\right]}^{-1}$$

In the case where the two end-state Δ*U* distributions are normal, the value of (*s*_1_β)^−1^ using eq 26 describes the range between the 0.02 Δ*U* quantile from ensemble 0 and the 0.98 Δ*U* quantile from ensemble 1.

## Methods

### Toy Systems

For the analytical demonstration and comparison of EQ and NEQ methods, we studied two types of toy systems: 1D harmonic oscillators and 2D-Ising models (Table 1).

**Table 1:** Five Toy Systems: Potential-Energy Function of Harmonic Oscillators (*U* ^HO^) is Described by Equation 27 ^a^

Systems	potential function	*U*_*A*_	*U*_*B*_	*s*_–10_
HO_1_	*U* ^HO^	*k* = 1, *x*_eq_ = −10	*k* = 1, *x*_eq_ = 10	–0.02493
HO_2_	*U* ^HO^	*k* = 1, *x*_eq_ = −10	*k* = 10, *x*_eq_ = 10	–0.00453
iHO	*U* ^HO^ + *U* ^bound^	*k* = −1, *x*_eq_ = – 10	*k* = −1, *x*_eq_ = 10	–0.02512
2D-Ising_1_	*U* ^Ising^	*h* = 1, *J* = 0.6	*h* = −1, *J* = 0.6	–0.02505
2D-Ising_2_	*U* ^Ising^	*h* = −1.5, *J* = 0.5	*h* = 3, *J* = 0.8	–0.01112

a An additional bounding potential *U* ^bound^ (eq 28) is applied on the iHO. The potential-energy function of a 2D-Ising model is described by eq 29. Columns *U*_*A*_ and *U*_*B*_ show the parameters used in the potential-energy function. *s*_–10_ shows the *s*-value used in each system according to eq 26 with α = −10.

#### 1D Harmonic Oscillators

The potential of 1D harmonic oscillators is described by an equilibrium position *x*_eq_ and a force constant *k*

27 $$U^{\mathrm{HO}}\left(x\right)=\frac{1}{2}k{\left(x-x_{\mathrm{eq}}\right)}^{2}$$

The first system (HO_1_) considers the transformation between two harmonic oscillators with equal force constant (*k*_*A*_ = *k*_*B*_ = 1) but shifted equilibrium positions (*x*_*A*_ = −10 and *x*_*B*_ = 10). The second system (HO_2_) consists of two harmonic oscillators with the same positional shift (*x*_*A*_ = −10 and *x*_*B*_ = 10) but different force constants (*k*_*A*_ = 1 and *k*_*B*_ = 10). In the third system (iHO), we consider inverted harmonic oscillators with the same positional shifts (*x*_*A*_ = −10 and *x*_*B*_ = 10) but negative force constants (*k*_*A*_ = *k*_*B*_ = −1). In this situation, a bounding potential *U* ^bound^ is applied to keep the system between *x*_*A*_ and *x*_*B*_

28 $$U^{\mathrm{bound}}\left(x\right)=\{\begin{array}{ll} \infty & \mathrm{if}\;x<x_{A}\;\mathrm{or}\;x>x_{B}\\ 0 & \mathrm{otherwise} \end{array}$$

#### 2D-Ising Models

The potential of a 2D-Ising model with an external magnetic field (*h*) and a spin–spin coupling strength (*J*) is described by

29 $$U^{\mathrm{Ising}}\left(S\right)=-\sum_{i}S_{i}\left(h+\sum_{j\in G\left(i\right)}JS_{j}\right)$$

where *S*_*i*_ is the *i*th spin in a 10 × 10 square spin–lattice, either up (+1) or down (−1), and G(i) represents the neighboring spins that are one grid away from spin *i*. The statistical state of an Ising model can be represented by the magnetization

30 $$m=\frac{1}{N}\sum_{i}S_{i}$$

where *N* = 100 is the number of spins in the system. The 2D-Ising model has a first-order transition when the external field *h* crosses zero at a temperature lower than the critical temperature^57^ $\left(T_{c}=\frac{2J}{k_{B}\ln\left(1+\sqrt{2}\right)}\approx 2.269\frac{J}{k_{B}}\right)$ , and thus represents a higher-dimensional phase-transition problem. System 2D-Ising_1_ (Table 1) involves flipping the external magnetic field, for which the net free-energy difference is zero. In contrast, system 2D-Ising_2_ involves changes in both the external field and coupling strength. Although no analytical solution is known for the 2D-Ising_2_ system, we assessed the performance by monitoring the convergence behavior of the free-energy estimate and the change in magnetization in the EQ and NEQ processes.

#### Simulation Details

Ten Metropolis-Hastings Monte Carlo (MC) simulations of the toy systems were performed with different random seeds. The configurations of the HO_1_, HO_2_, and iHO systems were initiated at *x* = 0 and a new configuration was proposed at each MC step with a maximum displacement of 0.5 along the *x*-axis. The 10 × 10-lattice configurations of the 2D-Ising systems were initiated randomly, and a new configuration was proposed at each MC step by flipping the directions of a randomly chosen spin. A new configuration was accepted or rejected following the Metropolis-Hastings criterion.^58^ The EQ simulations relied on 11 equidistant λ-states between zero and one with 5·10^5^ MC steps per λ-state and configurations saved every 50th step. The free-energy difference between the two end-states was evaluated with MBAR using the *mbar* module from the Python package pymbar.^17^ In the NEQ simulations, the coupling parameter λ was linearly scheduled from zero to one or from one to zero in the forward and backward transformations, respectively

31 $$\lambda_{k,\mathrm{forward}}=\frac{k}{\tau_{\mathrm{NEQ}}}$$

32 $$\lambda_{k,\mathrm{backward}}=1-\frac{k}{\tau_{\mathrm{NEQ}}}$$

The NEQ work was recorded at every step by

33 $$W_{k+1}=U_{k+1}\left(x_{k}\right)-U_{k}\left(x_{k}\right)$$

where *k* ∈ [0,τ_NEQ_] is the NEQ step index, *x*_*k*_ is the configuration from the last MC step, and *U*_*k*_(*x*_*k*_) and *U*_*k*+1_(*x*_*k*_) are the potential-energy values evaluated at *x*_*k*_ using the potentials at steps *k* and *k* + 1, respectively. 100 forward and backward transformations were performed from randomly drawn samples from ensembles *A* and *B*, respectively, with a maximum of 2·10^3^ NEQ steps per transformation. The dissipation of transformation *i* in the EI scheme was measured by the difference between the work and the equilibrium free-energy difference *W*_*d*_^*i*^ = *W* ^*i*^ – Δ*F*. As the energy offset shifts the potential energy by a constant *E*(λ = 1) at the end of the protocol (eqs 10 and 11), the mean dissipative work in the forward and backward directions in the EDS coupling scheme was computed by

34 $$\langle W_{d}\rangle_{\mathrm{forward}}=\langle W\rangle_{\mathrm{forward}}-\left[\Delta F_{\mathrm{AB}}-E\left(\lambda =1\right)\right]$$

and

35 $$\langle W_{d}\rangle_{\mathrm{backward}}=\langle W\rangle_{\mathrm{backward}}-\left[\Delta F_{\mathrm{BA}}+E\left(\lambda =1\right)\right]$$

Using the relation Δ*F*_*AB*_ + Δ*F*_*BA*_ = 0, the mean dissipative work from the bidirectional NEQ protocol is calculated by

36 $$\left\langle W_{d}\right\rangle =\frac{\langle W_{d}\rangle_{\mathrm{forward}}+\langle W_{d}\rangle_{\mathrm{backward}}}{2}=\frac{\langle W\rangle_{\mathrm{forward}}+\langle W\rangle_{\mathrm{backward}}}{2}$$

where the angular brackets indicate the mean over the forward and backward transformations. 100 frames were recorded evenly for each transformation for visualization. The free-energy difference between the two end-states was evaluated with CFT-BAR using the *bar* module from the Python package pymbar.^36^

### MD Simulations and Estimation of Solvation Free Energies

All molecular dynamics (MD) simulations were performed with the GAFF 1.8 force field^59^ and the TIP3P water model^60^ using the OpenMM 8.1 software.^61^ EQ simulations were performed using Langevin dynamics propagated with a CustomIntegrator combining the computation of EDS scaling factors with BAOA (or LFMiddle) discretization.^62^ The NEQ simulations were also performed using Langevin dynamics propagated with the time-symmetric BOAOB integrator from Leimkühler and Matthews,^63,64^ which we implemented as an OpenMM CustomIntegrator and adapted it to include the computation of the force scaling factor λ_EDS_(**r**, λ), such that no force or energy recalculation occurs outside of the integration loop.

All simulations were performed with an integration time step of 2 fs and a friction coefficient of 2 ps^–1^, and the pressure was kept at 1 atm using a Monte Carlo barostat. Full rigidity of water molecules was enforced with SETTLE^65^ and solute bond lengths were constrained with the CCMA algorithm.^66^ The long-range electrostatic interactions were handled using a reaction-field approach using atom-based truncation and a shifting function designed to prevent cutoff artifacts.^67,68^ The use of a reaction field permits the easy separation between the contributions of the nonbonded potential energy from the end-states and the environment,^68^ and the calculation of the absolute hydration free energy (AHFE) from a single simulation using the decoupling method.^69^ Additionally, previous comparisons of long-range treatments in liquid simulations suggest that reaction-field and PME-based protocols yield qualitatively comparable thermodynamic properties, suggesting that the performance of the method is unlikely to be affected by the specific choice of long-range electrostatic treatment.^70,71^ The Lennard-Jones (LJ) and electrostatic interaction cutoff distances were both set to 1.2 nm. To smoothen the transformation and avoid numerical instabilities, soft-core potentials were employed for both the charge and van der Waals interactions of the solute atoms, with the standard form as implemented in the AMBER18 software^72^ and the parameters α and β equal to 0.5 and 12 Å^2^, respectively.^73^ Two alternative soft-core formulations were also assessed, see Section S6 of the Supporting Information for details.

For all systems, starting coordinates of the solute molecules were retrieved from the FreeSolv database^46^ version 0.52. The molecules were solvated in a TIP3P^60^ water box, ensuring a padding of at least 1.2 nm between the solute surface and the nearest box wall. AHFE calculations were performed, in which the alchemical transformation turns the solute molecules from a fully interacting state (λ = 0) to a decoupled state (λ = 1). Equilibrium simulations, including all EQ windows as well as the end-state sampling of NEQ, were started from energy-minimized structures at the corresponding λ-value and equilibrated for 0.5 ns. EQ windows were sampled for 6 ns per window using 21 λ-values. For NEQ, 400 forward and 400 reverse switching trajectories in a range of τ_NEQ_ values between 1 and 10 ps were launched from randomly drawn samples collected from the end-state ensembles, which were sampled at equilibrium for 4 ns.

### Reference Values and Error Estimation from EQ Calculations

In order to establish reliable reference values, we first performed EQ calculations with 21 uniformly spaced λ-windows of 6 ns each, indexed as λ_*k*_ = *k*/20, for *k* = 0, 1,···,20. The set of AHFE values resulting from this large amount of sampling was used as accurate reference values for subsequent comparisons. Subsets of EQ data corresponding to *N* < 21 windows were obtained by taking samples from the N windows with index *k* matching $\mathrm{round}\left(20\cdot \frac{k^{\prime }}{\left(N-1\right)}\right)$ with *k*′ = 0,1,···, *N* – 1. Shorter simulation times were obtained from the full set of samples by splitting the data into blocks of subsamples corresponding to a specific simulation time. For example, to emulate a sampling of 0.6 ns based on the 6 ns simulation, one would group the samples in each window into 10 blocks of consecutive samples (6 ns/10 = 0.6 ns). Each block is then treated as a separate simulation, with its own free-energy estimate. A root-mean-square error (RMSE) was then calculated per molecule using the estimate of each block.

## Results and Discussion

### Analytical Demonstration Using Harmonic Oscillators as Toy Systems

To analyze the modulation of the local curvature in the coordinate domain and of the probability distribution in the Δ*U* domain, we investigated the effects of different *s* and *E*(λ) choices on the toy systems HO_1_, HO_2_ and iHO (Table 1), for which exact equilibrium properties can be computed analytically.

#### Harmonic Oscillator with Equal Force Constants

The system HO_1_ is illustrated graphically in Figure 1B. Figure 3A shows the probability distributions *p*(*x*) and *p̃*(Δ*U*) using *E*(λ) = 0 along with different *s*-values. In the EI coupling scheme (*s* = 0), the distributions retain the same Gaussian shapes while their means are shifting linearly with λ from *x*_*A*_ to *x*_*B*_. When *s* is negative, the means of the distributions no longer shift linearly with λ and the variance becomes narrower, indicating an increased curvature in the *x*-domain. Consistent with the discussion in the Theory section, the distributions in the Δ*U* domain are biased toward *E*(λ) (Figure 3A). When using *E*(λ) = *E*_lin_(λ) instead, the peaks of the coordinate probability distributions become smoother between the neighboring λ-intermediates and match those of the EI potential (Figure 3B). The choice of *E*_lin_(λ) also ensures the linearity of the potential minima with λ in a multidimensional HO when the two HOs share the same uncoupled force tensors. More details about how the EDS coupling scheme modulates the transformation in HOs are given in Section S2 in the Supporting Information.

**Figure 3:**
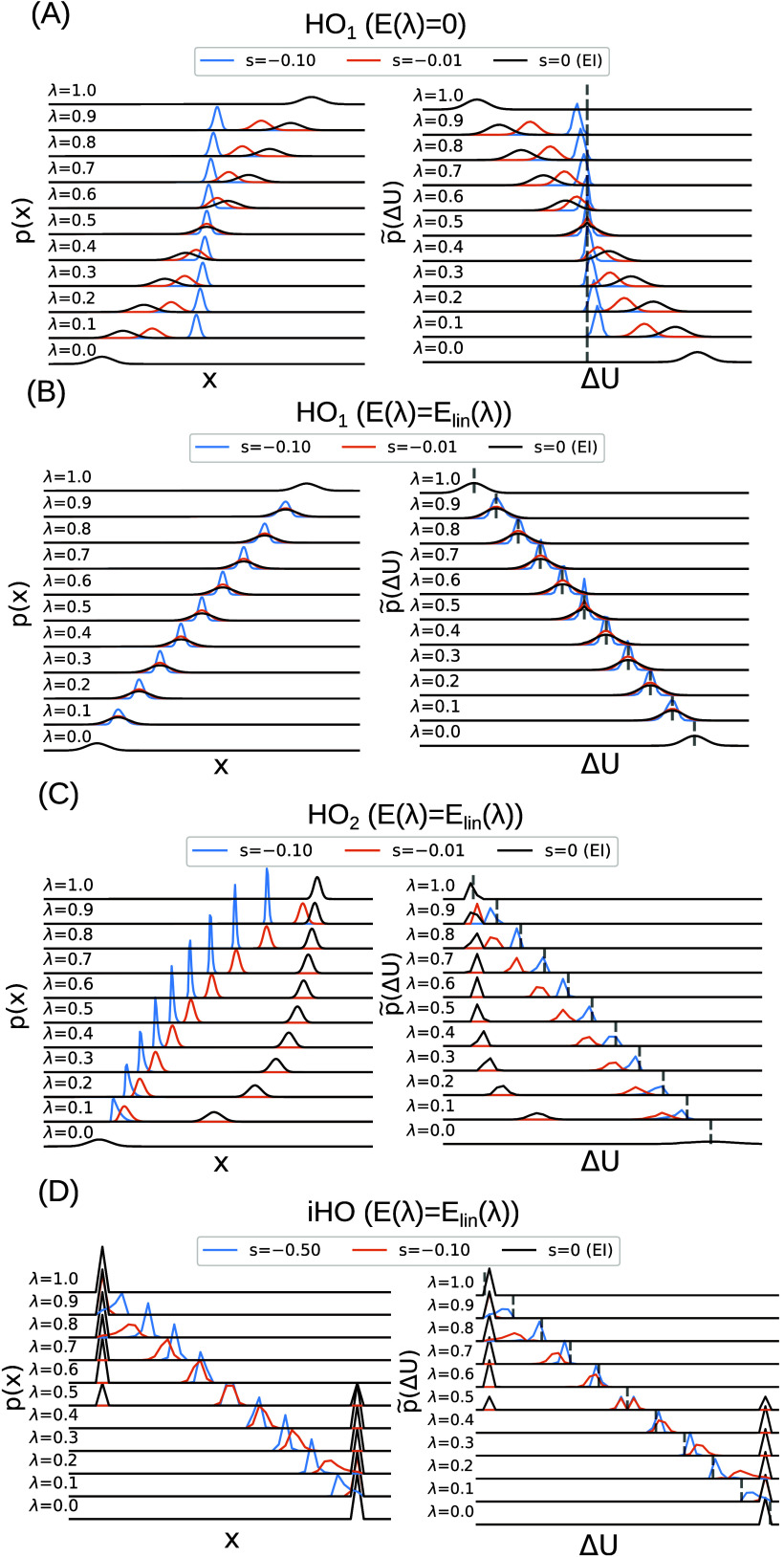
Modulation of the intermediate distributions *p*(*x*) and *p̃*(Δ*U*) by energy offsets *E*(λ) and negative *s*. (A): System HO_1_ without energy offset (*E*(λ) = 0). (B–D): Systems HO_1_, HO_2_, and iHO, respectively, with energy offset (*E*(λ) = *E*_lin_(λ)). The distribution of the EI coupling scheme (equivalent to *s* = 0 in eq 14) is shown in black and the distributions with negative *s*-values are shown in blue and orange.

#### Harmonic Oscillator with Different Force Constants

In the HO_2_ system, the EI coupling scheme shifts the probability peak from *x*_*A*_ to *x*_*B*_ faster at lower λ and slower at higher λ-values (Figure 3C). In contrast, a negative *s* with *E*(λ) = *E*_lin_(λ) smooths the transport profile in the *x*-domain along λ. The smoother transport of the distribution is explained by the altered distribution in the Δ*U* domain by *E*(λ). Although negative *s*-values move the mean of the Δ*U* distribution more smoothly along λ, they also narrow the distributions *p*(*x*) and *p̃*(Δ*U*) and therefore decrease the overlap between neighboring λ states when *s* becomes too negative.

#### Inverted Harmonic Oscillator with Bounding Potential

To illustrate the situation of a simple system with an intrinsically unstable transition path, we considered system iHO. In the EI coupling scheme, the curvature is negative everywhere inside the boundary and both *p*(*x*) and *p̃*(Δ*U*) accumulate at the boundary for every λ-value (Figure 3D). This is because the curvature along the *x*-axis, indicated by the second derivative of the potential-energy function, is negative everywhere between *x*_*A*_ and *x*_*B*_. In contrast, when a negative *s* is used together with *E*(λ) = *E*_lin_(λ), *p*(*x*) and *p̃*(Δ*U*) start to be populated between the boundary positions because of the increased curvature (as suggested by eq 16).

### Comparison of EQ and NEQ Methods on Toy Systems

The performance of the EQ and NEQ methods using the EDS versus the EI coupling scheme was compared for the five toy systems in Table 1. Note that only the results with the *s*-value corresponding to α = −10 in eq 26 (see Table 1 for the corresponding numerical values of *s*_–10_) will be shown in the main text, while other *s*-values (both positive and negative) are shown and discussed in Section S3 in the Supporting Information. In the following, we use the terms EQ-EI and EQ-EDS for the EQ free-energy estimators, and NE-EI and NE-EDS for NEQ free-energy estimators.

#### 1D Harmonic Oscillators

The advantages of the EDS coupling scheme are first demonstrated using the three toy systems HO_1_, HO_2_, and iHO. The EQ results are shown in Figure 4. In system HO_1_, the root-mean-square errors (RMSEs) of EQ-EI and EQ-EDS exhibit similar convergence (Figure 4A). As expected from the analytical results, the position distributions for the EDS intermediate states are narrower than those for EI (Figure 4A). In system HO_2_, EQ-EI gives an incorrect free-energy difference, whereas EQ-EDS reached an RMSE of about *k*_B_ *T* with 5·10^5^ MC steps per λ-value (Figure 4B). This is because there is little to no phase-space overlap between the first three λ-values in EI (Figure 4B). In contrast, the EDS intermediate states cover the position space more evenly, and create sufficient phase-space overlap for all windows (Figure 4B). In system iHO, EQ-EDS reached an RMSE of less than *k*_B_ *T* with 10^5^ steps per λ-value, whereas EQ-EI failed again to reach the correct solution because no samples were collected at intermediate positions for any of the λ-values (Figure 4C).

**Figure 4:**
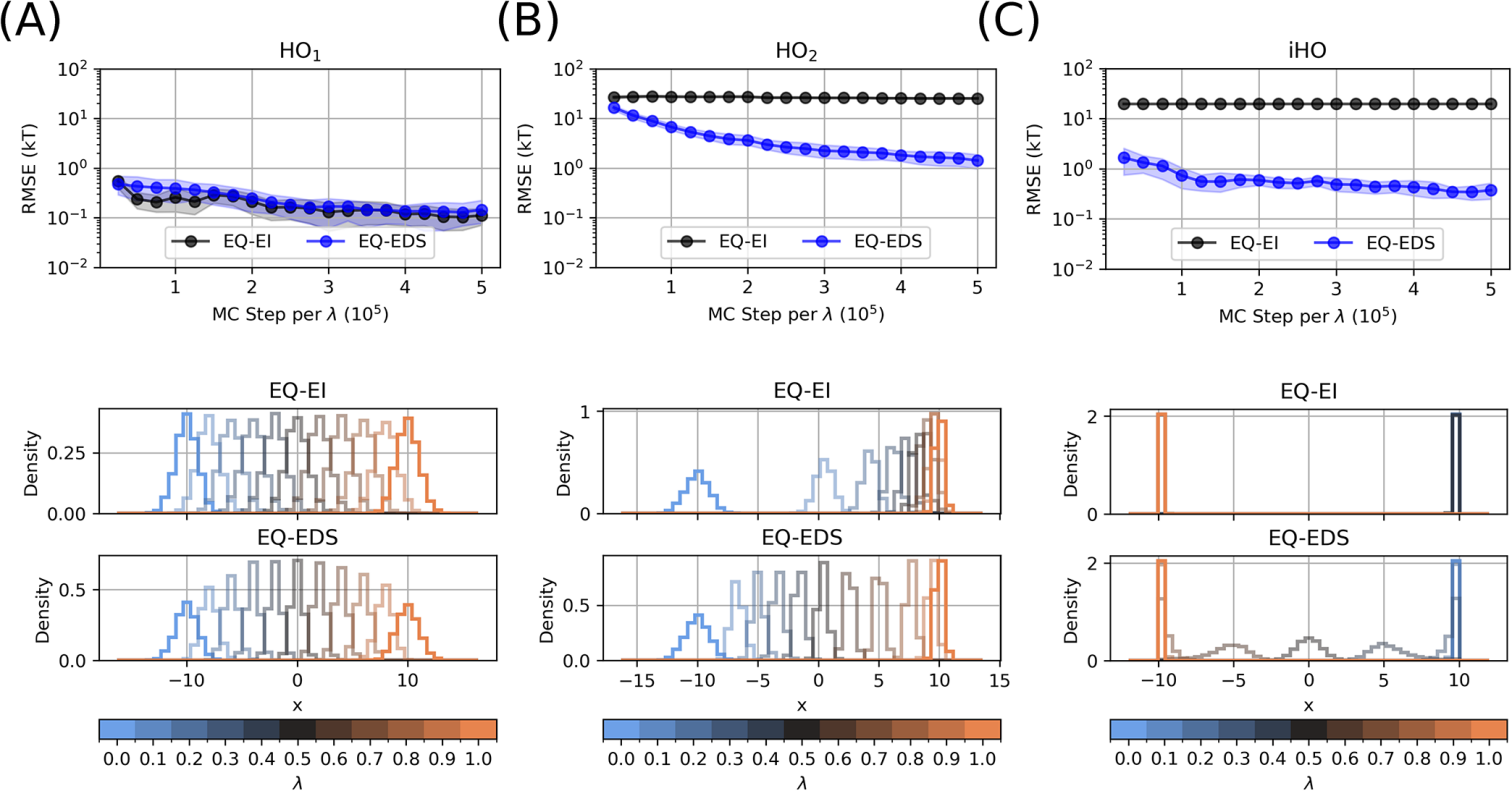
EQ simulations on systems HO_1_ (A), HO_2_ (B), and iHO (C). Upper panel: Root-mean-square error (RMSE) of the free-energy difference estimated from EQ sampling, computed relative to the analytical reference value, as a function of the number of MC steps used in each intermediate λ-value. The shaded area indicates the 95% confidence interval from bootstrapping the set of ten simulations. Lower panel: Coordinate distribution of the full EQ sampling data (5·10^5^ MC steps) with the EI and EDS coupling schemes. EQ-EDS simulations were carried out using *s* = *s*_–10_ as given in Table 1.

Next, we investigated the NEQ simulations. Similarly to the EQ simulations, NE-EI and NE-EDS show a comparable convergence for system HO_1_ (Figure 5A). Compared with NE-EI, the trajectory bundles generated from NE-EDS show a narrower distribution in the intermediate states (Figure 5A). In system HO_2_, the NEQ free-energy differences for both coupling schemes converge faster to the correct solution compared to EQ sampling (Figure 5B). Intermediate configurations are better sampled in NEQ compared to EQ when the transformation process is slow enough compared to the stochastic diffusion speed (compare Figures 4B and 5B) because NEQ methods connect source and target distributions through continuous trajectories (see Section S4 in the Supporting Information for further discussion). Nevertheless, the trajectories generated by NE-EDS are smoother than the ones from NE-EI, and the corresponding free-energy estimates are more accurate at every transformation speed. A similar improvement in the estimation of the free-energy difference is observed in the iHO system (Figure 5C). For each NE-EI transformation process, continuous trajectories connecting the two end-state distributions were generated when τ_NEQ_ was at least 3000 steps, allowing for an accurate free-energy estimate with an RMSE of around *k*_B_ *T*, which is a significant improvement over EQ-EI (compare Figures 4C and 5C). However, a strong hysteresis is observed, indicated by the gap between the forward and backward trajectories. In contrast, the NE-EDS trajectories generated show milder hysteresis and provide free-energy estimates with an RMSE of around 0.1 k_*B*_T when τ_NEQ_ was at least 10^4^ steps (Figure 5C).

**Figure 5:**
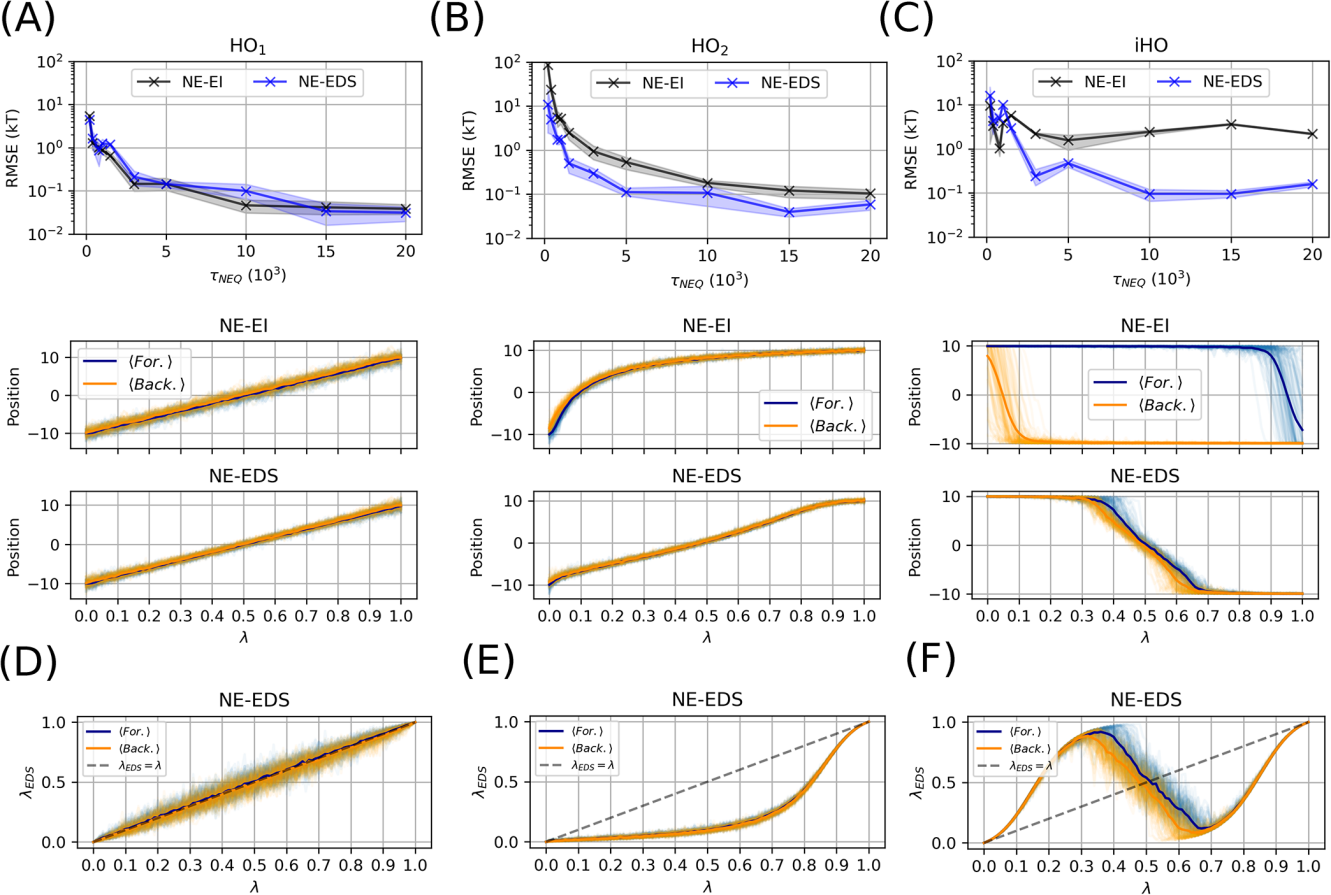
NEQ simulations on systems HO_1_ (A), HO_2_ (B), and iHO (C). Upper panel: RMSE of the free-energy difference estimated from NEQ sampling, computed relative to the analytical reference value, as a function of the number of sampling steps per NEQ trajectory. The shaded area indicates the 95% confidence interval from bootstrap resampling of the individual forward and backward work values. Lower panel: Visualization of the 100 forward (blue, λ: 0 → 1) and the 100 backward (orange, λ: 1 → 0) NEQ trajectories at τ_NEQ_ = 3000 steps. (D, E, F): Response of λ_EDS_ in the forward (blue) and backward (orange) NE-EDS transformation processes at τ_NEQ_ = 3000 steps on systems HO_1_ (D), HO_2_ (E), and iHO (F). The individual trajectories are depicted as transparent lines and the average quantities at each λ are shown as solid lines. NE-EDS simulations were carried out using *s* = *s*_–10_ given in Table 1.

Next, we investigated how NE-EDS smoothens the transformation by adapting λ_EDS_ to its instantaneous environment. Although λ_EDS_ behaves similarly to λ in the simple system HO_1_ (Figure 5D), it differs significantly from λ in the systems HO_2_ and iHO (Figure 5E and F). In system HO_2_, λ_EDS_ proceeds much slower than λ at the beginning of the forward process (Figure 5E), which balances the initial fast transition observed in the NE-EI scheme (Figure 5B). Interestingly, in system iHO, λ_EDS_ shows a fast-growing behavior to around 0.8 at the beginning of the forward process, followed by a decrease to around 0.2 between λ = 0.3 and λ = 0.7, during which the intermediate states were sampled (Figure 5C), and then again increases to 1 at the end (Figure 5F). The ability to adjust λ_EDS_ in a nonmonotonic manner according to instantaneous configurations shows the advantage of the EDS coupling scheme to allocate more computational resources to sample the transition regions.

In both NE-EI and NE-EDS, high RMSE values are observed in all systems when the NEQ transformation was performed too quickly (Figure 5A–C) and the system was driven far from equilibrium. The balance between the speed of the NEQ transformation and stochastic diffusion and its influence on the free-energy estimation is discussed further in Sections S4.1 and S4.2 in the Supporting Information. In addition, the uneven change in the phase space in system HO_2_ also leads to a higher dissipative work generation in the forward transformation than in the backward transformation (see Section S4.3 in the Supporting Information for further discussion).

#### 2D-Ising Models

To further demonstrate the strength of the EDS coupling scheme in more complex problems with an additional spatial dimension, we calculated the free-energy difference of two 2D-Ising models 2D-Ising_1_ and 2D-Ising_2_ (Table 1). In system 2D-Ising_1_, the free-energy difference estimated from EQ-EI did not converge to the correct value of zero, because it did not sample the magnetization states between −0.8 and 0.8 (Figure 6A). In contrast, the EQ-EDS estimator reached an RMSE of around 1 *k*_B_ *T* with 5·10^4^ MC steps per λ-value, and covered most magnetization values with 11 λ-values (Figure 6A). A similar behavior was also observed in the system 2D-Ising_2_, where the EQ-EI free-energy estimates were unreliable with poor phase-space coverage, whereas EQ-EDS showed better overlap between the neighboring λ-values, gradually converging to about −210 *k*_B_ *T* (Figure 6B).

**Figure 6:**
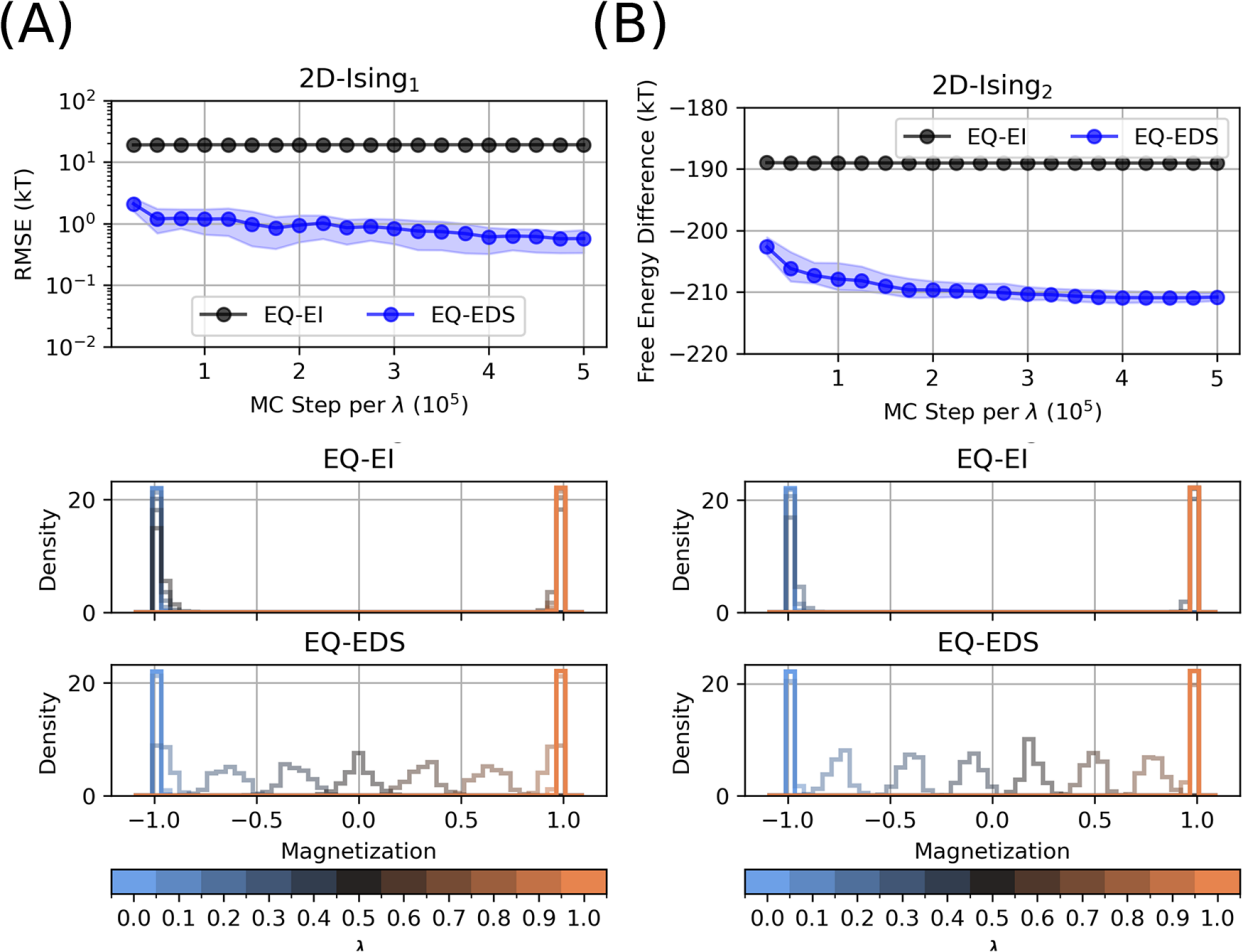
EQ MC simulations on systems 2D-Ising_1_ (A) and 2D-Ising_2_ (B). Upper panel: RMSE of the free-energy difference estimated from EQ sampling with respect to the number of MC steps used in each intermediate λ-value. Lower panel: Coordinate distribution of the full EQ sampling data (5·10^5^ MC steps) with the EI and EDS coupling schemes. EQ-EDS simulations were carried out using *s* = *s*_–10_ as given in Table 1.

For the NEQ simulations in system 2D-Ising_1_, NE-EI starts to reach the correct free-energy difference with an RMSE of around 2 *k*_B_ *T* at τ_NEQ_ = 2·10^4^ steps, which allowed the intermediate magnetization states to be sampled during the NEQ process, despite a large hysteresis (Figure 7A). In contrast, NE-EDS reached the same accuracy with shorter τ_NEQ_ and exhibited milder hysteresis in the NEQ trajectories. Although NE-EI slowly converged to −210 *k*_B_ *T* in the system 2D-Ising_2_ when τ_NEQ_ was at least 1.5·10^4^ steps, the free-energy estimate suddenly changed to −193 *k*_B_ *T* at τ_NEQ_ = 2·10^4^ steps (Figure 7B). This shows the fragility of NE-EI in applying the CFT-BAR estimator because of insufficient overlap in the forward and backward work distributions. This phenomenon is further described and characterized in Section S4.2 in the Supporting Information. In contrast, NE-EDS provided a more stable estimate of around −210 *k*_B_ *T* when τ_NEQ_ was at least 3·10^3^ steps with milder hysteresis in the NEQ transformation compared to NE-EI (Figure 7B). The ability of NE-EDS to more exhaustively sample the transition region can be attributed to the configuration-dependent λ_EDS_ in NE-EDS, which grew nonlinearly and spent most of the time sampling the configurations near the phase-transition region (Figure 7C–D).

**Figure 7:**
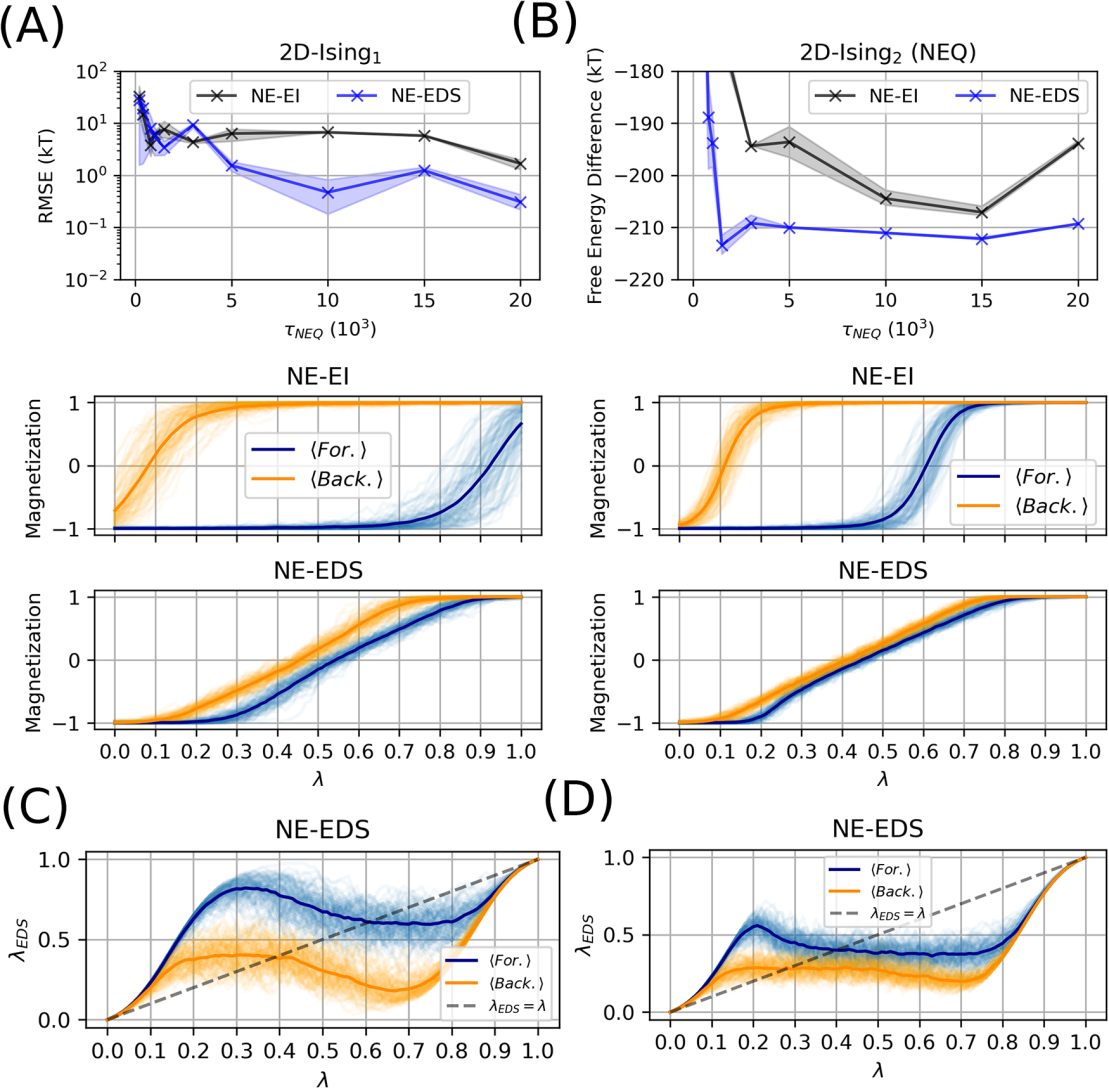
NEQ simulations on systems 2D-Ising_1_ (A) and 2D-Ising_2_ (B). Upper panel: RMSE of the free-energy difference estimated from NEQ sampling with respect to the sampling steps per NEQ trajectory. The shaded area indicates the 95% confidence interval from bootstrapping. Lower panel: Visualization of the 100 forward (blue, λ: 0 → 1) and the 100 backward (orange, λ: 1 → 0) NEQ trajectories at τ_NEQ_ = 3000 steps. (C, D): Response of λ_EDS_ in the forward (blue) and backward (orange) NE-EDS transformation processes at τ_NEQ_ = 3000 steps on systems 2D-Ising_1_ (C) and 2D-Ising_2_ (D). The individual trajectories are depicted as transparent lines and the average quantity at each λ shown as solid lines. NE-EDS simulations were carried out using *s* = *s*_–10_ as given in Table 1.

### Comparison on Absolute Hydration Free Energies

To assess the performance of the EDS coupling scheme for realistic applications, we calculated the absolute hydration free energies (AHFE) for the 642 small molecules in the FreeSolv data set^46^ and compared the results to the standard EI coupling in both EQ and NEQ protocols. We decided to use the longest EQ simulations (i.e., 21 λ-values with a 6 ns simulation time per window) as “ground truth” and not the experimental values as this factors out the effect of possible force-field errors. The correlation between these AHFE reference calculations and the corresponding experimental values is shown in Section S5.1 in the Supporting Information. For the comparison, the full 6 ns EQ trajectories were subsampled in order to mimic shorter effective simulation times (see Methods section).

Using the EQ approach, both EI and EDS converged smoothly toward the reference values as a function of simulation time (Figure 8A). When only 40–80 ps of six equally spaced λ-points were considered, the typical RMSE values were on the order of 2–3 kJ/mol, with a large variability across the set of molecules, highlighting the different degrees of difficulty of these transformations. When the simulation time was extended to 200–400 ps per λ-point, or when the number of points was increased from six to 21, the RMSE decreased substantially, reaching values around 1 kJ/mol. At 1–2 ns per window, both schemes produced errors consistently below 0.5 kJ/mol, approaching the statistical uncertainty of the reference calculation. Importantly, the two coupling schemes showed very similar performance throughout. Although the EDS scheme occasionally yielded slightly lower median errors, the differences were smaller than the intrinsic variability between molecules. Increasing the number of λ-points from six to 11 produced the expected improvement in overall accuracy, but did not qualitatively alter the comparison between EI and EDS. Thus, with EQ methods, the efficiency is dominated by the sampling duration and λ-spacing, with only a minor effect from the choice of coupling scheme.

**Figure 8:**
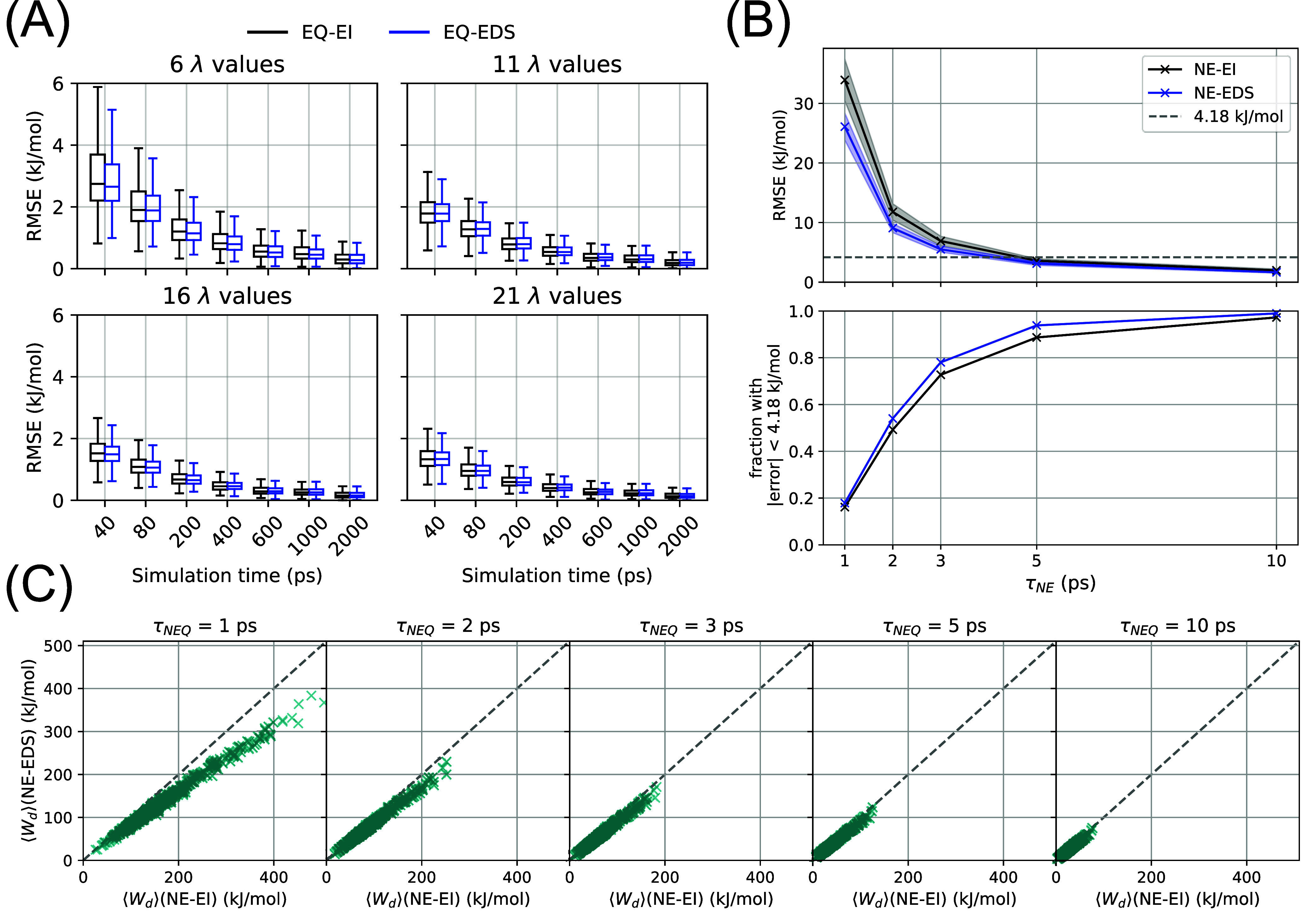
Computed absolute hydration free energies (AHFE) of the FreeSolv^46^ data set using the EI and EDS coupling schemes. (A): RMSE distributions of the estimates from the subsampled EQ simulation (outliers not shown for clarity) for different numbers of λ-points. (B): RMSE of the free-energy estimate with respect to τ_NEQ_ (top) and fraction of systems within chemical accuracy of the reference EQ free-energy difference with respect to τ_NEQ_ (bottom). (C): Comparison of the amount of dissipated work between the NEQ protocols for different τ_NEQ_ values.

The situation is very different when considering the NEQ approach. Comparing across the entire FreeSolv data set, NE-EDS outperformed NE-EI at all investigated transformation speeds in terms of both RMSE and the fraction of systems within chemical accuracy (i.e., absolute error <4.184 kJ/mol, or 1 kcal/mol) (Figure 8B). Notably, NE-EDS produces less dissipative work than NE-EI in almost all (98.5%) of the attempted NEQ transformations, especially for the fastest and most difficult transformations (Figure 8C). At short switching times (τ_NEQ_ = 1–3 ps), both NEQ protocols suffered from substantial dissipative work production, leading to large RMSE values in excess of 20 kJ/mol for the shortest switching time and highly variable performance across different solutes (Figure 8B and C). In several cases, the work distributions exhibited very poor overlap between forward and reverse directions, leading to unstable free-energy estimates. At τ_NEQ_ = 5 ps, both methods approached chemical accuracy for most systems (Figure 8B). Finally, at the longest investigated switching time (τ_NEQ_ = 10 ps), the two protocols converged to the EQ reference within statistical error, as work production became low enough for most systems. (Figure 8B and C). These studies highlight that the benefits of EDS are most substantial under NEQ conditions and when the transformations are difficult, as most of the error reduction is observed for the molecules with the highest deviation (see Section S5.2 in the Supporting Information).

Since the difficulty of the AHFE calculation lies in creating a solute-shaped cavity in water, the amplitude of dissipative work production correlates well with molecular weight (Pearson correlation coefficient ρ = 0.93). In addition, near perfect correlations can be observed between the produced dissipative work and several size-related 2D molecular descriptors,^74^ such as chi0v^75^ and labuteASA^76^ (Pearson coefficient ρ = 0.99 and 0.98, see Section S5.3 in the Supporting Information for more details).

To further characterize the smaller dissipative work production by NE-EDS at the molecular level, we investigated the NEQ process in individual compounds. Figure 9 illustrates the AHFE calculation of the heaviest molecule in the FreeSolv data set (decachlorobiphenyl, compound *mobley_5456566*) at the slowest NEQ transformation process (τ_NEQ_ = 10 ps), where high dissipative work was produced for both NE-EI and NE-EDS. The main difference between the two schemes arises during the backward switching (recoupling), where NE-EDS produces systematically less work (⟨*W*_*d*_⟩_backward_ = 90 and 68 kJ/mol for NE-EI and NE-EDS, respectively, Figure 9A,B). This points to a specific molecular bottleneck. In the soft/decoupled region (λ ∈ [0.5,1]), NE-EI tends to keep the solute too soft to immediately repel solvent as the interactions are turned on. As a result, solvent remains within the solute volume, and the cavity is created at considerably lower λ-values compared to the forward process. This asymmetric response is directly visible in the average nearest-neighbor distance *r* ®_NN_ of the solute particles. In NE-EI, *r* ®_NN_ stays small over much of the backward process and increases only at relatively low λ-values, indicating a late and abrupt cavity creation (Figure 9E). Such a late solvent expulsion is mechanically irreversible and therefore costly in work, explaining the heavier backward tail in the work distribution for NE-EI (Figure 9A).

**Figure 9:**
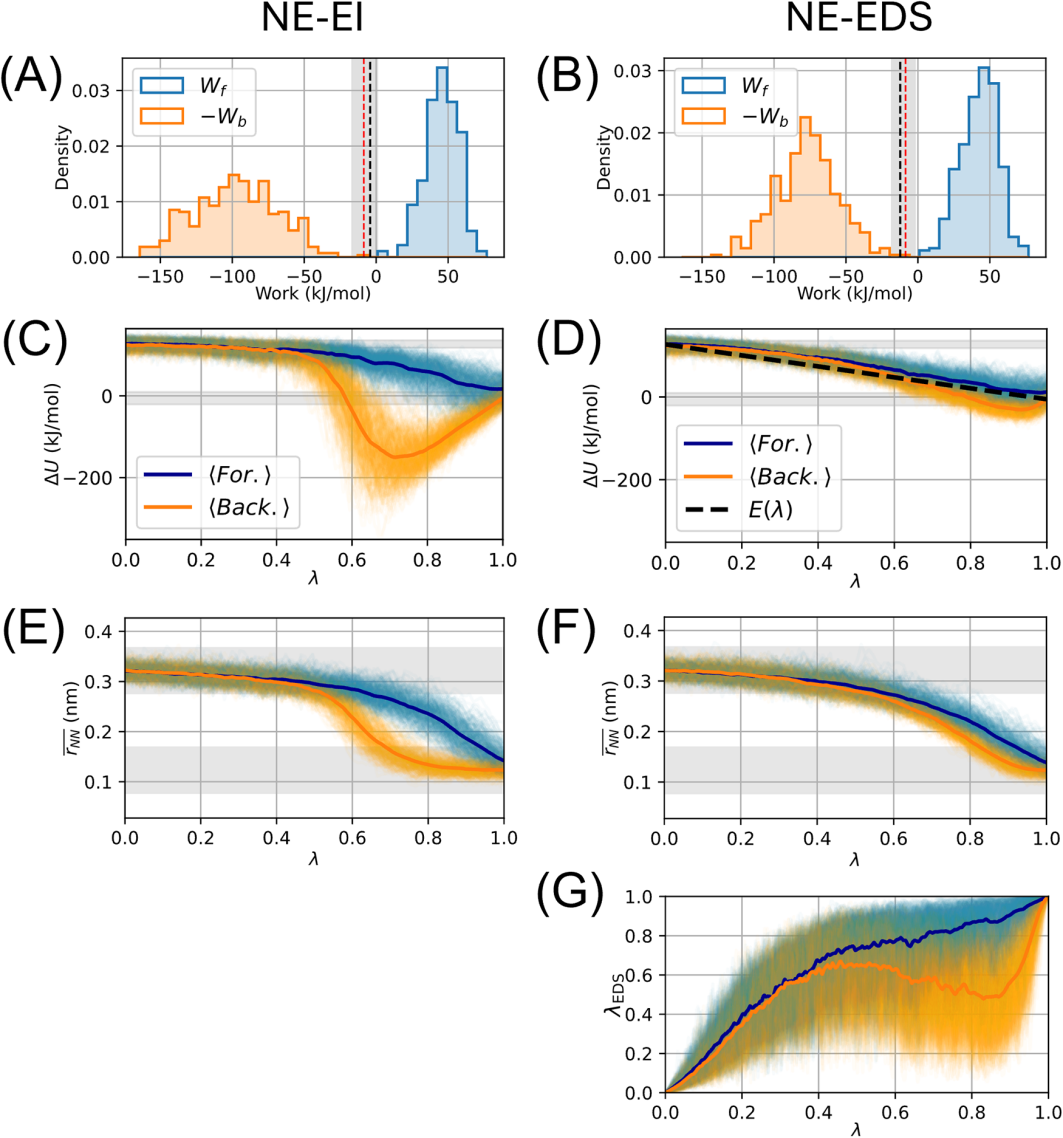
NEQ transformation of the heaviest molecule in FreeSolv (decachlorobiphenyl, *mobley_5456566*) at τ_NEQ_ = 10 ps. (A, B): Work distributions from forward (blue) and backward (orange) transformations, reference ΔG (red dashed line), estimated ΔG (black dashed line), and 95% CI (shaded area). (C, D): End-state energy difference and energy offsets (black dashed line). (E, F): Average nearest-neighbors distance between solute atoms and the nearest solvent atoms. (G): Effective λ_EDS_ of the forward and backward trajectories. The ± 1 standard deviation ranges of ΔU and *r*_NN_ sampled from the end-states simulations are shown with shaded areas (C–F).

NE-EDS mitigates this failure mode by adapting the effective coupling to the instantaneous configuration. This mechanism is reflected by the behavior of the Δ*U*(**r**) distributions along the protocol. In NE-EI, the forward and backward Δ*U*(**r**) distributions strongly disagree in this high-λ region, consistent with a pronounced configuration–coupling mismatch (i.e., the system lags behind the protocol). In NE-EDS, this forward–backward discrepancy is reduced because the protocol includes the linear offset *E*(λ), which pulls the sampled Δ*U*(**r**) values toward a common target across directions (Figure 9D). When a configuration would otherwise lead to delayed cavity reopening (i.e., solvent remains too close while recoupling), Δ*U*(**r**) becomes inconsistent with the target set by *E*(λ). The configuration-aware variable λ_EDS_ then increases to strengthen solute–solvent forces earlier, repulsing the solvent preemptively (Figure 9G). Consistent with this interpretation, the backward cavity creation becomes smoother in NE-EDS, as shown by the more gradual increase of *r* ®_NN_ (Figure 9F). In general, we found that the reduced dissipative works in NE-EDS are mainly observed in the backward switching direction (see Section S5.4 in the Supporting Information).

From a methodological perspective, these results suggest that a substantial fraction of the dissipation in this alchemical transformation arises from concentrating solvent-cavity formation into a relatively narrow interval of the alchemical coordinate. In principle, this could be alleviated by redesigning the underlying soft-core functional form to distribute cavity formation more evenly (e.g., by turning on the repulsive LJ core more gradually, or adjusting the relative pace of LJ and electrostatic coupling). Similar considerations have motivated previous NEQ alchemical protocols in which the electrostatic and LJ interactions were regularized to improve bidirectional work-distribution overlap.^40^ However, such protocol tuning is necessarily transformation-dependent and it is generally nontrivial to define soft-core parameters that perform robustly across chemically diverse solutes and environments. By contrast, NE-EDS targets the underlying cause of the hysteresis—the configuration-coupling mismatch—through its adaptive effective coupling, and is therefore complementary to, and largely independent of, the specific alchemical functional form used in the coupled Hamiltonians. Furthermore, additional investigations into alternative soft-core schemes indicate that NE-EDS can robustly handle less well-tuned choices of soft-core parameters, considerably increasing the relative RMSE improvement over NE-EI from 1.9 kJ/mol to as much as 11.2 kJ/mol (Section S6 in the Supporting Information).

The smoother cavity creation in NE-EDS due to its configuration-aware λ_EDS_ is generally observed across most of the molecules investigated in the study, with more pronounced gains for larger molecules. When growing a more polar but smaller molecule (e.g., 1,1,1-trifluoro-2-propanol, compound *mobley_628086* in the FreeSolv database), NE-EDS also creates the cavity preemptively; however, the reduction in dissipative work is less pronounced, presumably due to its smaller size (Section S7 in the Supporting Information). Indeed, polarity-related features, such as topological polar surface area (TPSA),^77^ showed only weak correlations with dissipative work (Pearson coefficient ρ = 0.40, Section S5.3 in the Supporting Information).

The specific contributions from the electrostatic and LJ interactions can be isolated by performing NEQ simulations of two model transformations: dipole inversion and particle insertion. These reveal that the modulation of λ_EDS_ is mainly due to cavity-creation processes rather than local electrostatic perturbation, which ultimately leads to the greater reduction of dissipative work observed in NE-EDS (more details in Section S8 in the Supporting Information).

## Conclusions

The EDS method was initially introduced as a coupling scheme to sample multiple ensembles in a single simulation. The present work is the first to systematically investigate and validate the use of a negative smoothing parameter *s* in pairwise alchemical transformations. We demonstrate that this specific application transforms the EDS scheme into a powerful and flexible tool for free-energy calculations, offering superior control over the construction of the intermediates between two end-states.

Our findings consistently show that by employing a negative *s*-parameter, the EDS coupling scheme effectively smoothens the phase-space transformation, i.e., prevents abrupt jumps in the relative end-state energies (Δ*U*(**r**) distributions) during the transformation process. This increases the local curvature of the potential-energy landscape, thereby preventing phase transitions and end-point catastrophes seen in simulations relying on a traditional EI coupling scheme. The benefits of this approach are clearest in systems where EI coupling introduces abrupt changes in phase-space overlap. In the harmonic and Ising test systems, EDS was found to consistently improve the stability and accuracy of both EQ and NEQ free-energy estimates. In AHFE calculations, we did not observe a significant advantage of EDS over EI under EQ sampling, where both coupling schemes converged similarly to the reference values. However, EDS presents a clear advantage in the NEQ regime, attributed to the configuration-aware λ_*EDS*_, which regularizes solute insertion rates based on the mismatch between the instantaneous Δ*U*(**r**) and the energy offset *E*(λ), thus reducing dissipation and improving the free-energy estimates. In particular, we found that the “difficulty” of AHFE estimation is almost perfectly correlated with the molecular size of the solutes, whose insertion rates are regularized by λ_EDS_.

In summary, the EDS coupling scheme provides a simple, fundamental, yet significant advantage for free-energy calculations. Without additional computational expense, it leads to smoother and more stable alchemical transformations. We anticipate that this work will create future opportunities for other advanced free-energy methods.

## Supplementary Material



## Data Availability

Simulation inputs and analysis scripts are freely available on the GitHub repository: https://github.com/rinikerlab/SmoothTransformationWithEDS.

## Notes

Discussion of minimal-energy path; effects of different *s* values and NEQ switching speeds; dissipative work in the forward and backward directions; detailed analyses of the AHFE results, including correlations with RDKit 2D descriptors; case study of a small and polar compound, and simple alchemical transformations (PDF)
