# Supplementary material for: Smoother Alchemical Transformations via Enveloping Distribution Sampling for Free-Energy Estimation
Source: J Chem Theory Comput. 2026 Jul 13;22(14):7297–312. doi: 10.1021/acs.jctc.6c00581 (PMC13422002; doi:10.1021/acs.jctc.6c00581)
Supplement: Supplementary file 1 [file ct6c00581_si_001.pdf]

SUPPORTING INFORMATION
Smoother Alchemical Transformations via Enveloping Distribution Sampling for Free-Energy Estimation

Shu-Yu Chen,[a,b] Enrico Ruijsenaars,[a,b] Philippe H. Hünenberger,[a] and Sereina Riniker*[a]

[a] *Department of Chemistry and Applied Biosciences, ETH Zürich, Vladimir-Prelog-Weg 2, 8093 Zürich, Switzerland. E-mail: sriniker@ethz.ch*

[b] *Authors contributed equally.*

Contents

S1 Minimal-Energy Path in the EI and EDS Coupling Schemes **S2**

S2 Minimal-Energy Path and Curvature in Harmonic Oscillators with the Same Force Constant **S3**

S3 EQ-EDS and NE-EDS with Different α-Values **S5**

S4 NEQ Simulations with Different Switching Speeds **S7**
S4.1 Far from Equilibrium with Fast NEQ switching S7
S4.2 Work Distributions at Different Switching Speeds S9
S4.3 Work Dissipation in Forward and Backward Directions S16
S4.4 Estimation Quality is More Sensitive to the Switching Speed than the Number of NEQ Trials S17

S5 Detailed Analysis of AHFE Benchmarking **S19**
S5.1 Comparison of Reference Computed AHFE Values with Experiment S19
S5.2 AHFE Error Comparison Between Coupling Schemes S19
S5.3 Correlation Between Dissipative Work in AHFE NEQ Simulations and 2D Physicochemical Descriptors S20
S5.4 Asymmetry of Dissipative Work S22

S6 Sensitivity of NEQ AHFE Results to the Choice of Soft-Core Parameters **S23**

S7 Case Study of the Small Polar Molecule *mobley_628086* in NEQ Simulations **S25**

S8 Simple Alchemical Transformations **S26**
S8.1 Dipole Inversion S27
S8.2 Particle Insertion S28

S1 Minimal-Energy Path in the EI and EDS Coupling Schemes

All points $\{\mathbf{r}_m(\lambda)\}$ on a minimal-energy path (MEP) are characterized by a vanishing first derivative with respect to all coordinates:

$$\frac{\partial U(\mathbf{r},\lambda)}{\partial r_i}\bigg|_{\mathbf{r}_m} = 0. \tag{1}$$

Taking the first derivative of Eq. 1 with respect to λ gives:

$$\frac{\partial}{\partial\lambda}\left(\frac{\partial U(\mathbf{r}_m(\lambda),\lambda)}{\partial r_i}\right) = \frac{\partial^2 U}{\partial\lambda\partial r_i} + \sum_j^d \left(\frac{\partial^2 U}{\partial r_i \partial r_j}\frac{\partial r_m^j}{\partial\lambda}\right) = [\mathbf{g} + \mathbf{H}\frac{d\mathbf{r_m}}{d\lambda}]^i = 0, \tag{2}$$

where d is the number of degrees of freedom in the system, with cross gradient $g_i = \frac{\partial^2 U}{\partial\lambda\partial r_i}$ and the Hessian matrix $H_{ij} = \frac{\partial^2 U}{\partial r_i \partial r_j}$. Inverting the Hessian matrix from Eq. 2 gives the evolution of the MEP along λ:

$$\frac{d\mathbf{r}_m}{d\lambda} = -\mathbf{H}^{-1}\cdot\mathbf{g}\bigg|_{\mathbf{r}_m}, \tag{3}$$

where the dot notation indicates an inner product. In the EI and EDS coupling schemes, the cross gradients are:

$$\mathbf{g}_{EI}(\lambda) = [(1-\lambda)\mathbf{g}_A + \lambda\mathbf{g}_B] + (\mathbf{f}_A - \mathbf{f}_B) \tag{4}$$

$$\mathbf{g}_{EDS} = [(1-\lambda_{EDS})\mathbf{g}_A + \lambda_{EDS}\mathbf{g}_B] + \lambda_{EDS}(1-\lambda_{EDS})\left[\frac{1}{\lambda(1-\lambda)} - s\beta(\frac{\partial U_A}{\partial\lambda} + \frac{dU_B}{d\lambda} - \frac{dE(\lambda)}{d\lambda})\right](\mathbf{f}_A - \mathbf{f}_B). \tag{5}$$

Equation 3 shows that the EDS coupling scheme could also alter the MEP along λ. In the low $s\beta(\Delta U(\mathrm{r}) - E(\lambda))$ regime, one can approximate the Hessian and the cross gradient of the EDS coupling scheme in a perturbation form:

$$\mathbf{H}_{EDS} \approx \mathbf{H}_{EI} - s\beta\lambda(1-\lambda)(\mathbf{f}_A - \mathbf{f}_B)(\mathbf{f}_A - \mathbf{f}_B)^T \tag{6}$$

$$\mathbf{g}_{EDS} \approx \mathbf{g}_{EI} - s\beta\lambda_{EDS}(1-\lambda_{EDS})\left(\frac{\partial U_A}{\partial\lambda} + \frac{dU_B}{d\lambda} - \frac{dE(\lambda)}{d\lambda}\right)(\mathbf{f}_A - \mathbf{f}_B). \tag{7}$$

To preserve the same MEP as in the EI coupling scheme, $\mathbf{r}_m^{EDS} = \mathbf{r}_m^{EI} = \mathbf{r}_m$ in the low s regime, one can substitute Eqs. 6 and 7 into the last part of Eq. 2:

$$\begin{aligned}\mathbf{g}_{EDS} + \mathbf{H}_{EDS}\frac{d\mathbf{r_m}}{d\lambda} &\approx \left(\mathbf{g}_{EI} + s\beta\lambda\left(\frac{\partial U_A}{\partial\lambda} + \frac{dU_B}{d\lambda} - \frac{dE(\lambda)}{d\lambda}\right)\mathbf{f}_B\right) + \left(\mathbf{H}_{EI} - s\beta(\frac{\lambda}{1-\lambda})\mathbf{f}_B\mathbf{f}_B^T\right)\frac{d\mathbf{r_m}}{d\lambda} \\ &= s\beta\lambda\mathbf{f}_B\left(\frac{\partial U_A}{\partial\lambda} + \frac{dU_B}{d\lambda} - \frac{dE(\lambda)}{d\lambda} - \frac{1}{1-\lambda}\mathbf{f}_B^T\frac{d\mathbf{r_m}}{d\lambda}\right) \\ &= 0,\end{aligned} \tag{8}$$

where the MEP condition $(1-\lambda)\mathbf{f}_A\Big|_{\mathbf{r}_m} = -\lambda\mathbf{f}_B\Big|_{\mathbf{r}_m}$ is used. Equation 8 implies the condition for the energy offset in the EDS coupling scheme to resemble the EI MEP:

$$\frac{dE(\lambda)}{d\lambda} = \frac{\partial U_A}{\partial\lambda} + \frac{dU_B}{d\lambda} - \frac{1}{1-\lambda}\mathbf{f}_B^T\frac{d\mathbf{r_m}}{d\lambda}. \tag{9}$$

S2 Minimal-Energy Path and Curvature in Harmonic Oscillators with the Same Force Constant

For d-dimensional harmonic oscillators, the potential energy, force, and Hessian can be described in matrix form:

$$U^{HO}(\mathbf{r}) = \frac{1}{2}(\mathbf{r} - \mathbf{r_{eq}})^T \mathbf{K}(\mathbf{r} - \mathbf{r_{eq}}), \tag{10}$$

$$\mathbf{F}(\mathbf{r}) = -\mathbf{K}(\mathbf{r} - \mathbf{r_{eq}}), \tag{11}$$

$$\mathbf{H}(\mathbf{r}) = \mathbf{K}, \tag{12}$$

where $\mathbf{r_{eq}} \in \mathbb{R}^d$ is the equilibrium position and $\mathbf{K} \in \mathbb{R}^{d \times d}$ is the matrix of positive-definite force constants. As an example of an uncoupled system, the force-constant matrix is diagonal:

$$\mathbf{K} = \begin{bmatrix} k^1 & 0 & 0 & \cdots & 0 \\ 0 & k^2 & 0 & \cdots & 0 \\ 0 & 0 & k^3 & \cdots & 0 \\ \vdots & \vdots & \vdots & \ddots & \vdots \\ 0 & 0 & 0 & \cdots & k^d \end{bmatrix}. \tag{13}$$

When applying the EI coupling scheme, the MEP in the ith degree of freedom is:

$$(r_m^{EI})^i(\lambda) = \frac{(1-\lambda)k_A^i r_A^i + \lambda k_B^i r_B^i}{(1-\lambda)k_A^i + \lambda k_B^i}, \tag{14}$$

where k_A^i and r_A^i are the force constant and the equilibrium position of system A, and k_B^i and r_B^i are the force constant and the equilibrium position of system B. With the choice of the same force constant $k_A^i = k_B^i = k^i$, the energy difference along the MEP between the two states becomes linear in λ and equal to the linear energy offset $E_{lin}(\lambda)$ (see main text):

$$U_B(\mathbf{r}_m^{EDS}, \lambda) - U_A(\mathbf{r}_m^{EI}, \lambda) = \sum_i (\frac{1}{2} - \lambda) k^i (r_A^i - r_B^i)^2 = E_{lin}(\lambda). \tag{15}$$

This indicates that with the choice of $E(\lambda) = E_{lin}(\lambda)$, λ_{EDS} always equals λ at any selection of s. The corresponding value of s^* is:

$$s^*(\mathbf{r}, \lambda)|_{\mathbf{r}=\mathbf{r}_m} = \frac{1}{\beta}\left[\lambda(1-\lambda)(\sum_i k^i (x_B^i - x_A^i)^2)\right]^{-1} \tag{16}$$

for all i. Given that $\lambda(1-\lambda) \in [0, 0.25]$, a negative curvature is induced when $s < \frac{4}{\beta}\left[\sum_i k^i (x_B^i - x_A^i)^2\right]^{-1}$. In Figure S1A, forward and backward MEP are shown for $k^i = 1 k_B T$ and $x_B^i - x_A^i = 20$, for which the critical s-value $s^*(\mathbf{r}_m, \lambda, E)|_{\lambda=0.5}$ is 0.01. When a higher s-value is chosen, hysteresis is observed, regardless of whether $E(\lambda)$ is applied. When an s-value lower than 0.01 is chosen (Figure S1B), no hysteresis is observed and the EI-MEP (linear shift with λ) is preserved when a linear energy offset is applied. When s is close to the critical value 0.01, the Hessians become zero at $\lambda = 0.5$.

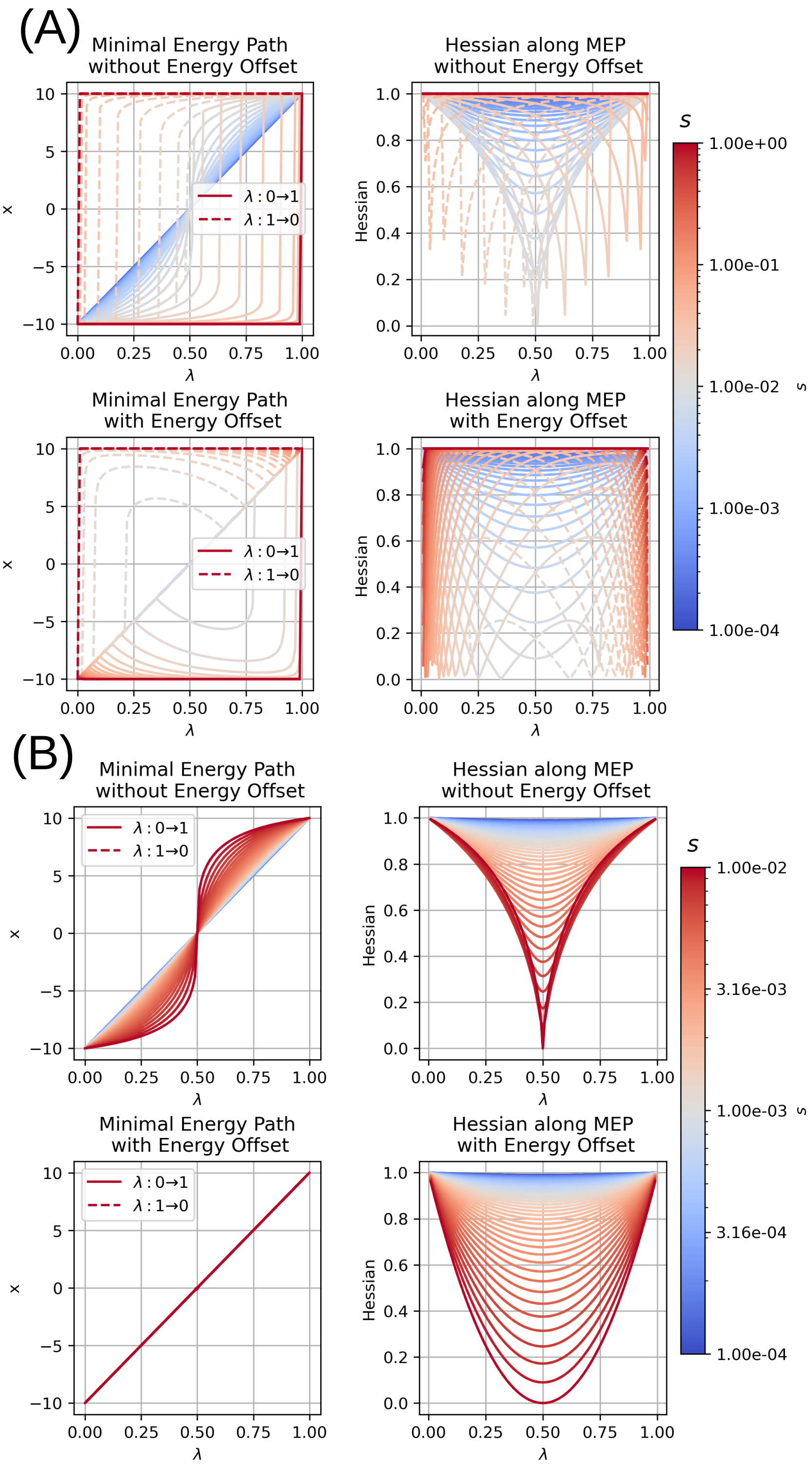

Figure S1: Preservation of the minimal-energy path (MEP) of the EI coupling scheme using the EDS coupling scheme in the system of 1D harmonic oscillators with the same force constant. (A): MEP of the EDS coupling scheme without an energy offset (upper panel) and with linear energy offset (lower panel) for $s \in [10^{-4}, 1]$. (B): MEP and the Hessian of the EDS coupling scheme without energy offset (upper panel) and with linear energy offset (lower panel) for $s \in [10^{-4}, 10^{-2}]$. MEPs derived from the forward direction (minimized from $x = -10$) and the backward direction (minimized from $x = 10$) are shown as solid and dashed lines, respectively.

S3 EQ-EDS and NE-EDS with Different α-Values

While only the results with $s = s_{-10}$ were presented in the main text, other s-values corresponding to $\alpha = [10, 2, -2, -20, -50]$ were also tested in the five toy systems to evaluate the dependence on the choice of s in terms of RMSE (Figure S2) and dissipative work (Figure S3). For the highest s-value ($\alpha = 10$), the MBAR solver did not converge in any EQ simulation due to the large barrier caused by the EDS coupling scheme (Figure S2). In the NEQ simulations, EDS with the positive s-values ($\alpha = 2$ and $\alpha = 10$) consistently generated the largest dissipative work in all systems (Figure S3), leading to poor free-energy convergence (Figure S2). Except for the EQ simulations in system 2D-Ising$_2$, where MBAR failed to converge at $\alpha = -50$, MBAR converged much more slowly for $\alpha = -20$, no significant differences were observed when using different negative s-values.

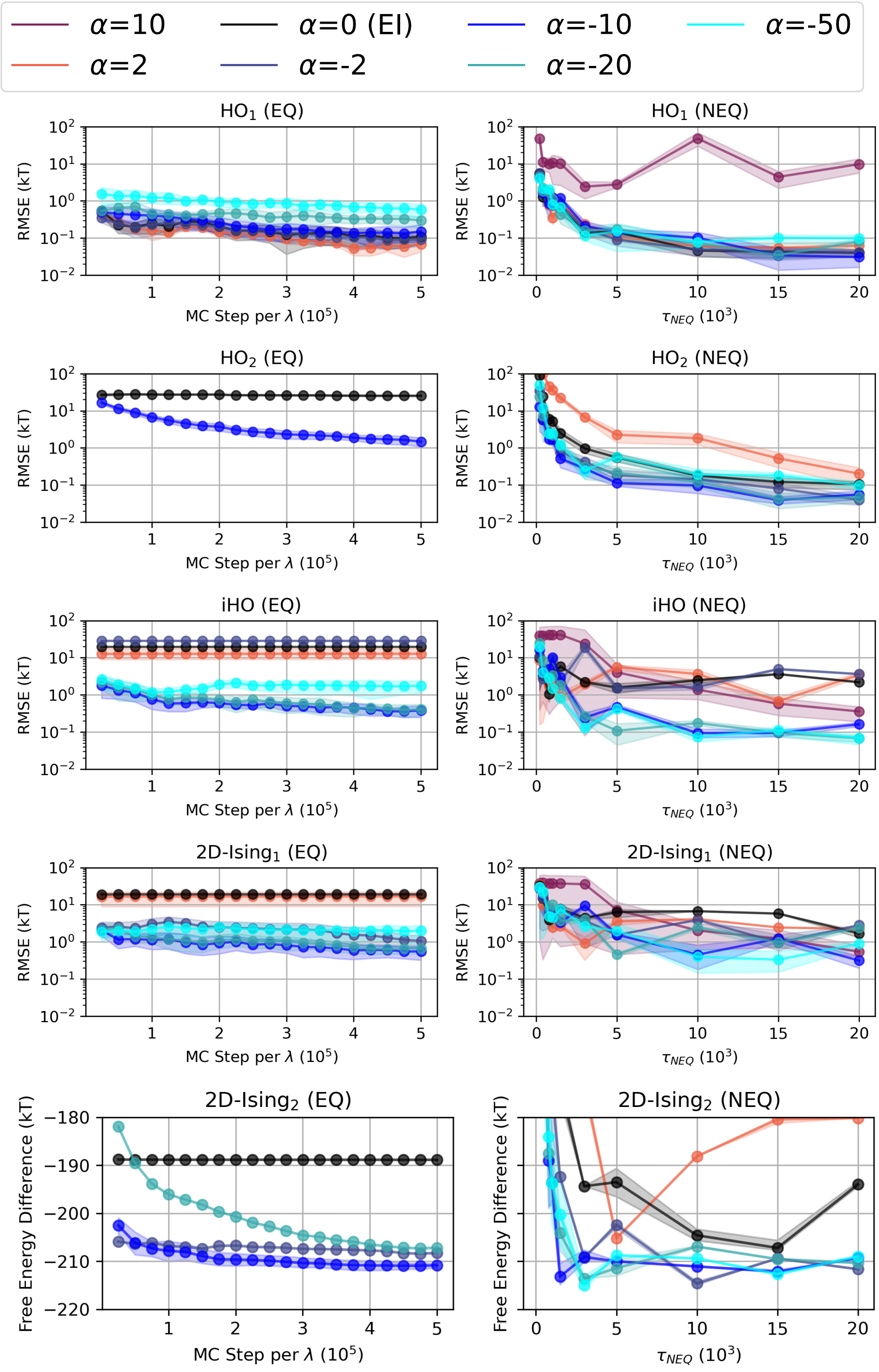

Figure S2: Root-mean-square error (RMSE) obtained in the five toy systems with the EQ and NEQ methods using different s-values (controlled by α). The shaded area indicates the 95% confidence interval from bootstrapping. Data curves are not shown for simulations that fail to converge the MBAR or CFT-BAR equation.

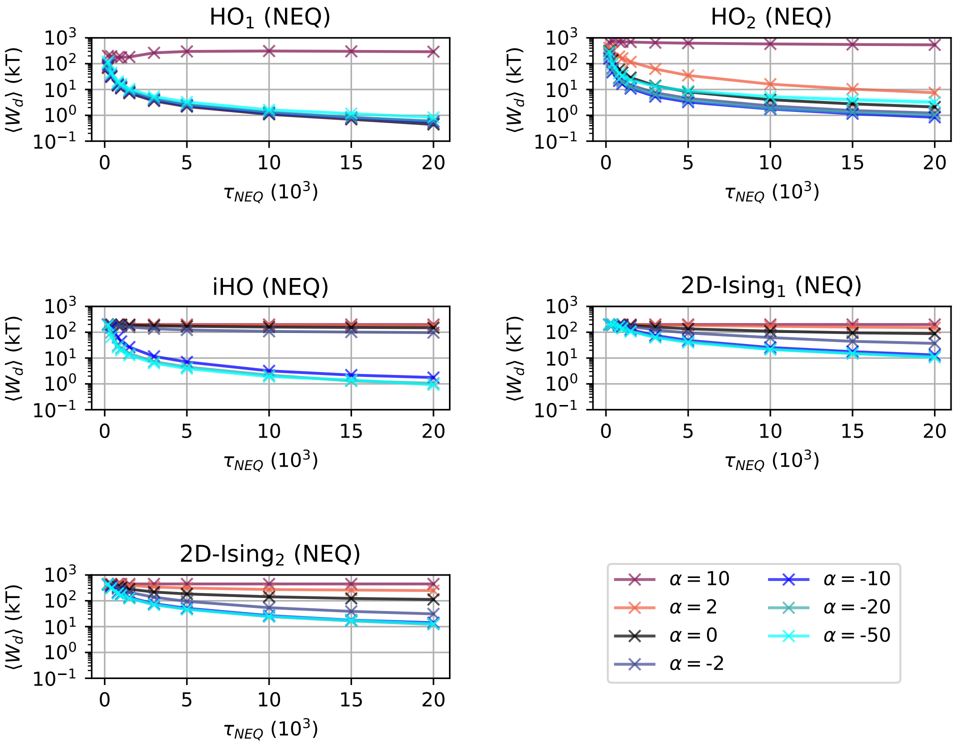

Figure S3: Mean dissipative work in the five studied systems for different NEQ shooting steps (τ_{NEQ}) and different s-values (controlled by α). The shaded area indicates the 95% confidence interval from bootstrapping. Data are not shown for simulations that fail to converge the MBAR or CFT-BAR equation.

S4 NEQ Simulations with Different Switching Speeds

In the main text, NEQ simulations with fast switching speeds generally yield a significantly higher RMSE for the toy systems. In this section, we show the trajectories of the faster transformations ($\tau_{NEQ} = 400$ steps, Figures S4 and S5) and the work distributions with different λ-switching speeds (Figures S6-S10).

S4.1 Far from Equilibrium with Fast NEQ switching

In Section S1, we discussed the local MEP that describes the local changes in the minimal-energy coordinate upon the perturbation of a change in λ. Ideally, if the λ-switching is slow enough, the system would have enough time to relax to the new minimal-energy coordinate via stochastic diffusion and therefore "catch up" with the MEP. However, when the switching speed is too fast compared to the diffusion process, the system can gradually lag behind the MEP, which can be directly observed through hysteresis in the coordinate trajectories. The hysteresis, as an indication of irreversibility, produces higher dissipative work and, thus, leads to poor free-energy estimates.

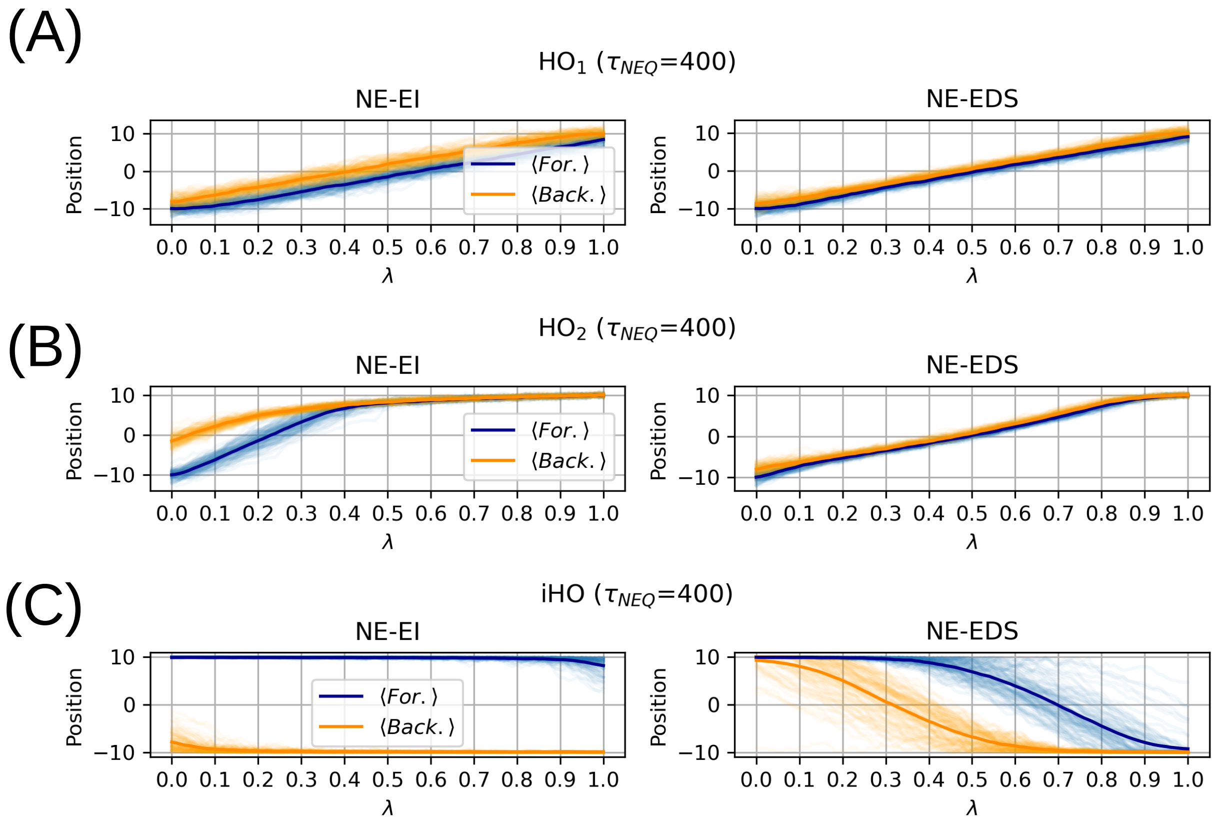

Figure S4: Visualization of the 100 forward (blue, $\lambda : 0 \to 1$) and 100 backward (orange, $\lambda : 1 \to 0$) NEQ trajectories with faster transformations at $\tau_{NEQ} = 400$ steps in systems: (A) HO_1, (B) HO_2, and (C) iHO using NE-EI (left panels) and NE-EDS (right panels).

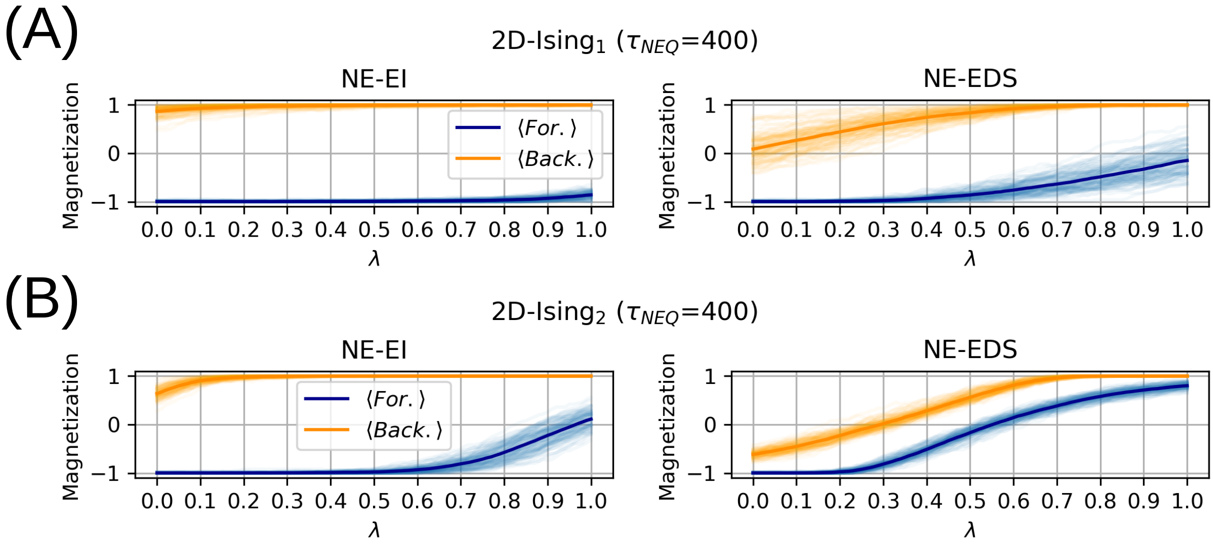

Figure S5: Visualization of the 100 forward (blue, $\lambda : 0 \rightarrow 1$) and 100 backward (orange, $\lambda : 1 \rightarrow 0$) NEQ trajectories with fast transformations at $\tau_{NEQ} = 400$ steps in systems: (A) 2D-Ising$_1$ and (B) 2D-Ising$_2$ using NE-EI (left panels) and NE-EDS (right panels).

At τ_{NEQ} = 400 steps, both NE-EI and NE-EDS showed only mild hysteresis in system HO$_1$ (Figure S4A). However, for systems HO$_2$ and iHO, NEQ simulations started to show hysteresis and yielded unreliable free-energy estimates (Figures S4B and C). We note that the harmonic-oscillation systems only differ in the force constant but share the same equilibrium positions. Therefore, the distance required for near-equilibrium diffusion is the same (from $x = -10$ to $x = 10$). While NE-EI suffers from the fast-changing MEP in systems HO$_2$ and iHO, NE-EDS effectively modifies the MEP and the local curvature for smoother transformations that are easier for the diffusion process to catch up. In the two 2D Ising models, shorter transformation times also mean that the configurations, represented by magnetization, do not reach the end-state equilibrium magnetization, e.g., +1 or -1, at the end of the transformation, showing the high irreversibility of the transformations.

S4.2 Work Distributions at Different Switching Speeds

In NEQ simulations, the free-energy difference can be obtained by solving the CFT-BAR equation with the forward and backward work values collected from multiple NEQ switching experiments:

$$\sum_{i}^{n_f} \frac{1}{1 + \frac{n_f}{n_b} e^{\beta(W_i - \Delta F_{AB}^{CFT})}} = \sum_{j}^{n_b} \frac{1}{1 + \frac{n_b}{n_f} e^{\beta(W_j + \Delta F_{AB}^{CFT})}}, \tag{17}$$

where n$_f$ and n_b are the number of NEQ switching trajectories in the forward and backward directions, respectively. The Fermi-like functions on both sides of the equation get the most statistical information from the overlap region of the forward and negative backward work distributions, which occurs near $W \approx \Delta F_{AB}^{CFT}$. In other words, having the estimated ΔF_{AB}^{CFT} in the overlap region of the two work distributions yields the highest statistical efficiency.

Figures S6-S10 show the work distributions of the NEQ simulations in the five toy systems with transformation time $\tau_{NEQ} = [800, 3000, 10000, 20000]$ steps. In general, when the switching becomes slower,

the means of the work distributions become closer, as quantified by the smaller mean dissipative work $\langle W_d \rangle = \frac{\langle W_f \rangle + \langle W_b \rangle}{2}$ (Figure S3), while the variance becomes smaller.

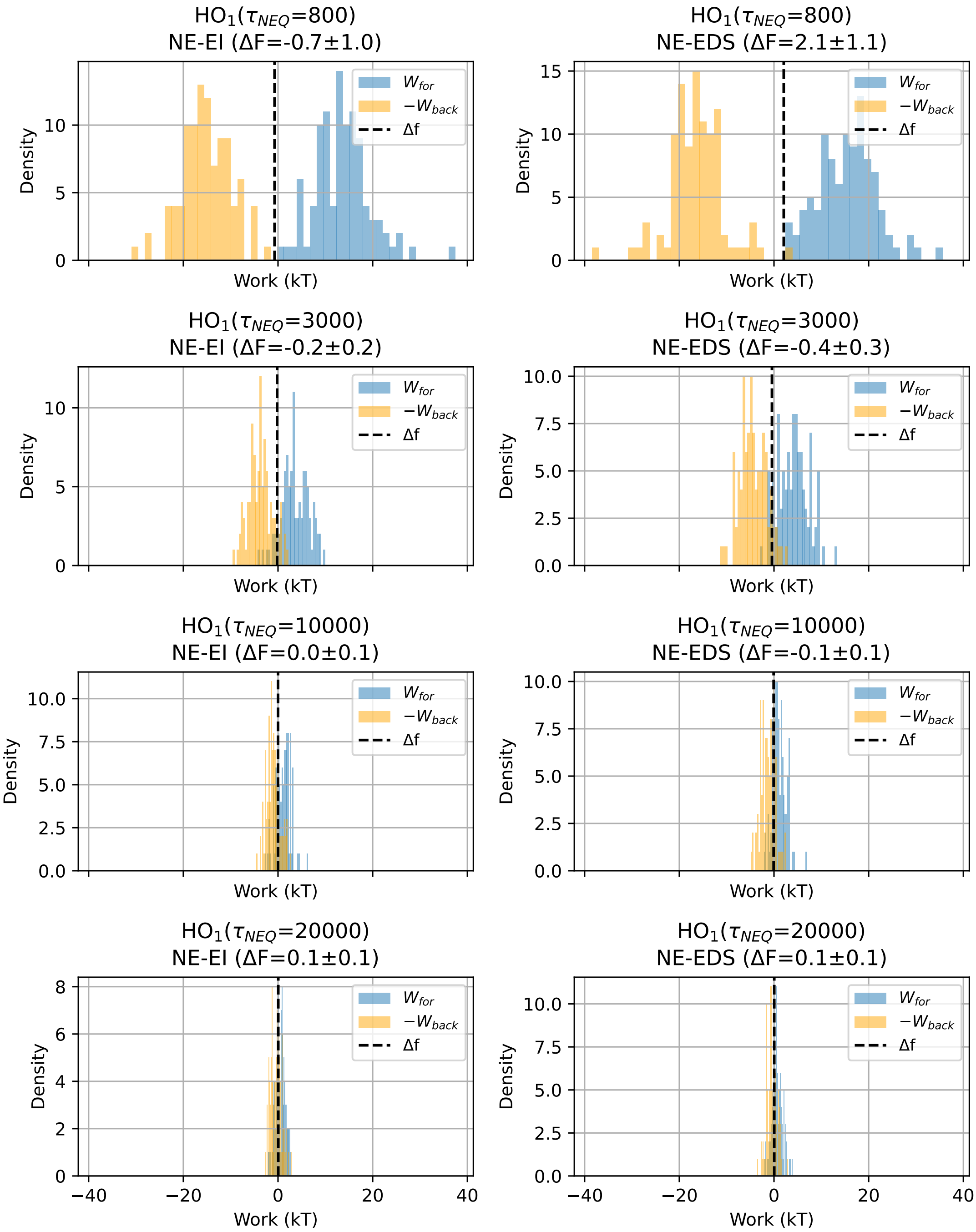

Figure S6: Forward (blue) and backward (orange) work distributions collected in NEQ simulations in system HO_1 with different NEQ transformation speeds using EI and EDS coupling schemes. The estimated free-energy difference is shown in the title in k_BT units and plotted with the black dashed line. The analytical free-energy difference is zero.

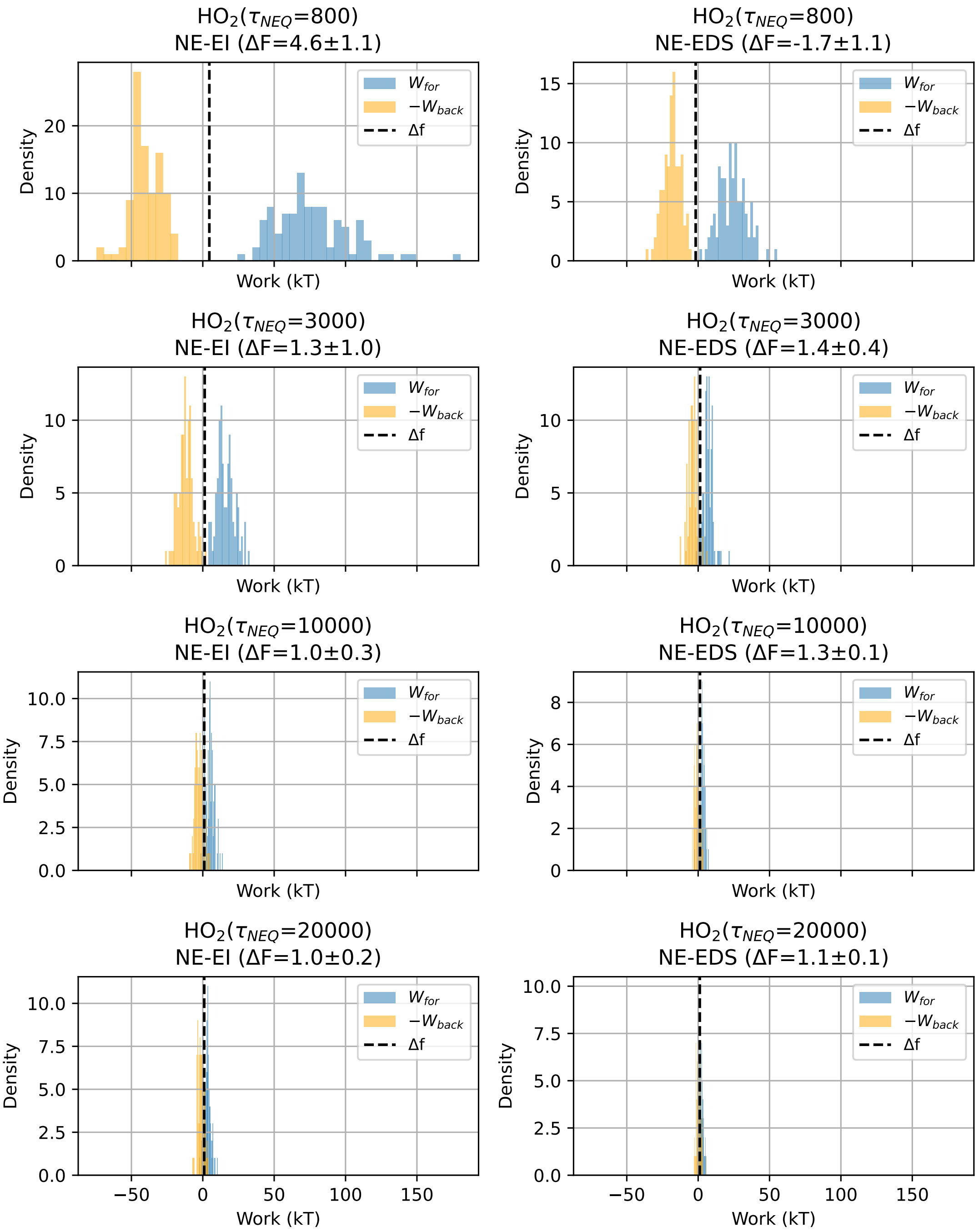

Figure S7: Forward (blue) and backward (orange) work distributions collected in NEQ simulations in system HO_2 with different NEQ transformation speeds using EI and EDS coupling schemes. The estimated free-energy difference is shown in the title in k_BT units and plotted with the black dashed line. The analytical free-energy difference is 1.153 k_BT.

When sufficient overlap between the distributions is obtained, several work values contribute efficiently to the statistically optimal CFT-BAR free-energy estimate. In contrast, when there is no overlap, the esti-

mated free-energy difference relies heavily on the few lowest work values and is therefore very sensitive to outliers. This is because most of the work values are much higher than the estimated ΔF values and therefore do not contribute to the CFT-BAR equation because of the vanishing term $\frac{1}{1+e^{\beta(W-\Delta F)}}$ in Eq. 17. Examples of the low overlap in the work distributions due to an ill-adapted NEQ protocol are shown for systems iHO (Figure S8), 2D-Ising$_1$ (Figure S9), and 2D-Ising$_2$ (Figure S10). Compared to the NE-EDS protocols, where the two distributions begin to overlap at longer τ_{NEQ} and the estimated free-energy differences slowly converge to certain values, NE-EI protocols fail to form overlap and consequently show large fluctuations as τ_{NEQ} is increased. In particular, the single outlier in the NE-EI backward simulation of 2D-Ising$_2$ at $\tau_{NEQ} = 2 \cdot 10^4$ steps (the orange outlier in Figure S10) largely biases the estimate toward a higher value.

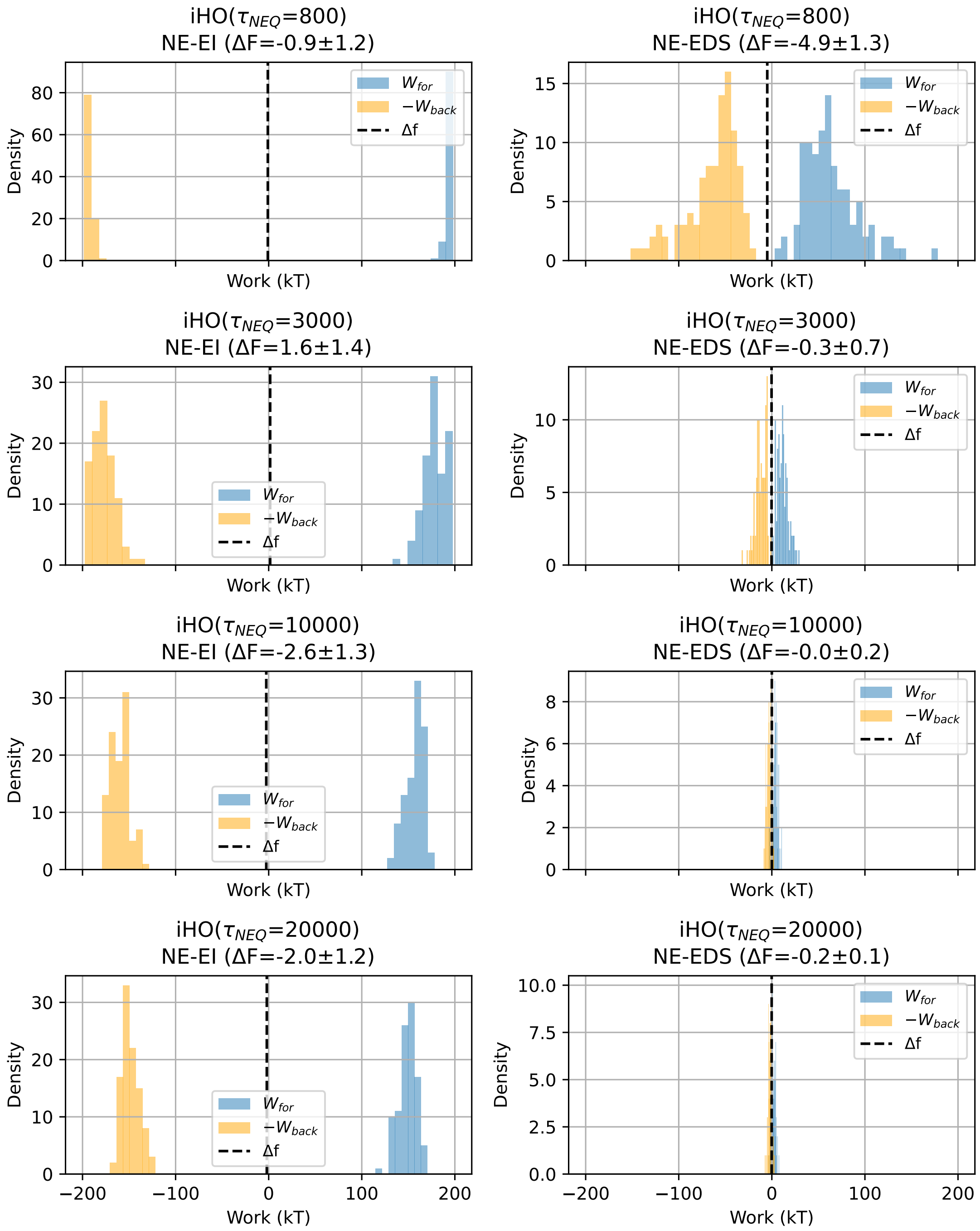

Figure S8: Forward (blue) and backward (orange) work distributions collected in NEQ simulations in system iHO with different NEQ transformation speeds using EI and EDS coupling schemes. The estimated free-energy difference is shown in the title in k_BT units and plotted with the black dashed line. The analytical free-energy difference is zero.

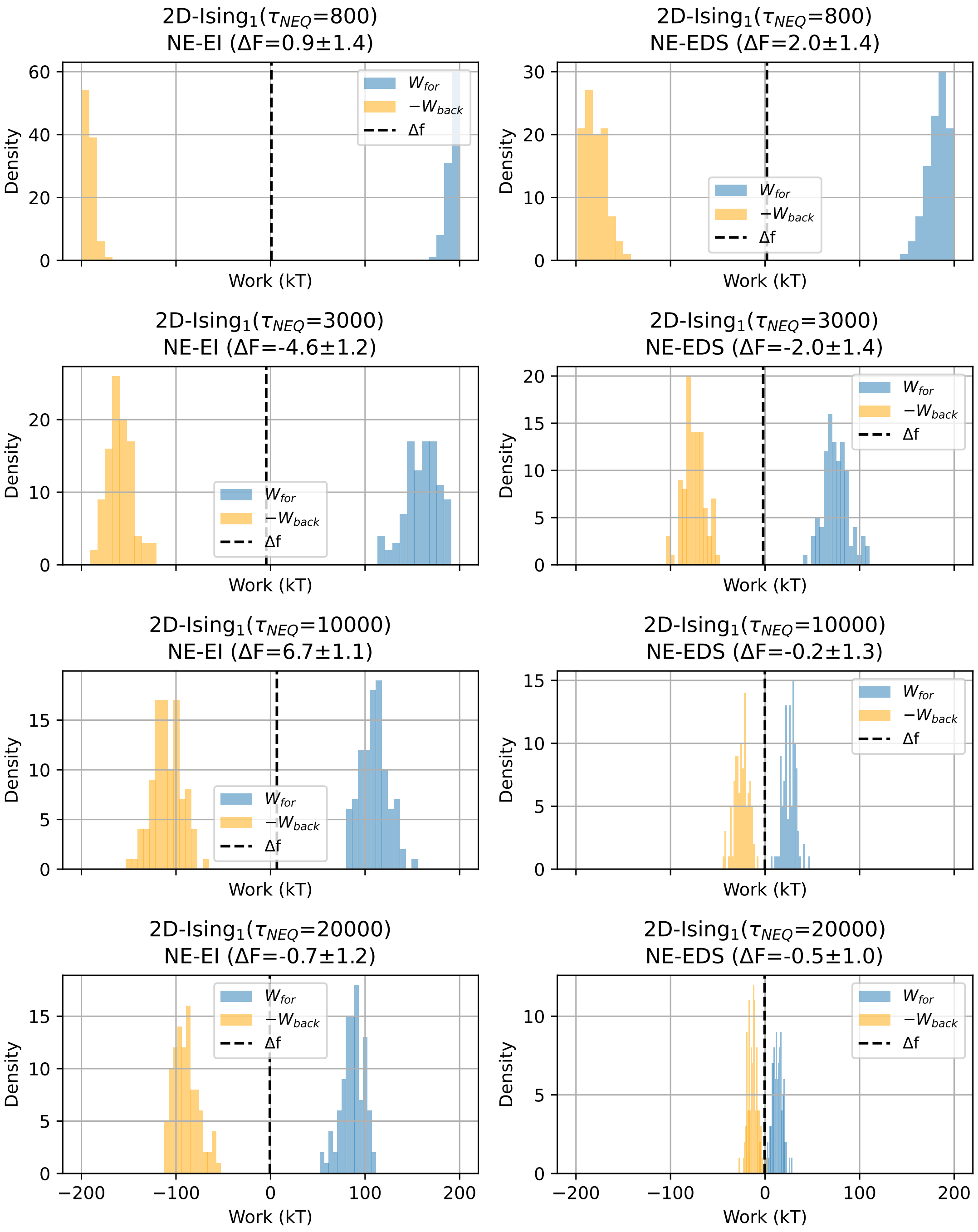

Figure S9: Forward (blue) and backward (orange) work distributions collected in NEQ simulations in system 2D-Ising$_1$ with different NEQ transformation speeds using EI and EDS coupling schemes. The estimated free-energy difference is shown in the title in k$_B$T units and plotted with the black dashed line. The analytical free-energy difference is zero.

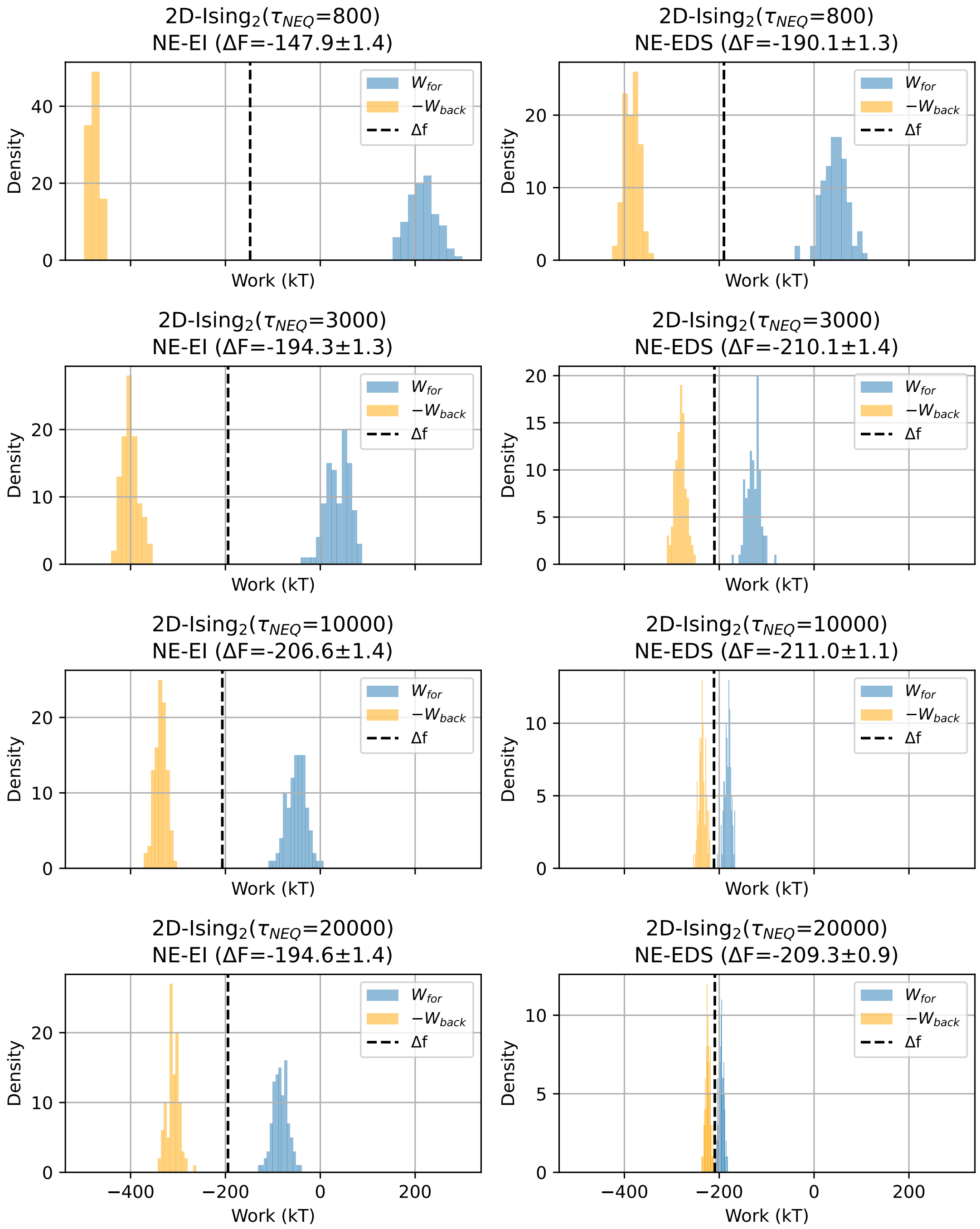

Figure S10: Forward (blue) and backward (orange) work distributions collected in NEQ simulations in system 2D-Ising$_2$ with different NEQ transformation speeds using EI and EDS coupling schemes. The estimated free-energy difference is shown in the title in k_BT units and plotted with the black dashed line. The analytical free-energy difference is unknown.

S4.3 Work Dissipation in Forward and Backward Directions

Figure S11 shows the mean dissipative work measured in the forward and backward transformations. For the transformation protocols that symmetrically change the phase-space probability distributions during the transformation process, such as HO_1, iHO, and 2D-$Ising_1$, the forward and backward transformations generate similar dissipative work (Figure S11). In contrast, for asymmetric systems such as HO_2 and 2D-$Ising_2$, where the change in phase-space is more dramatic in the low-λ regions, the forward transformation generates higher dissipative work than the backward transformation.

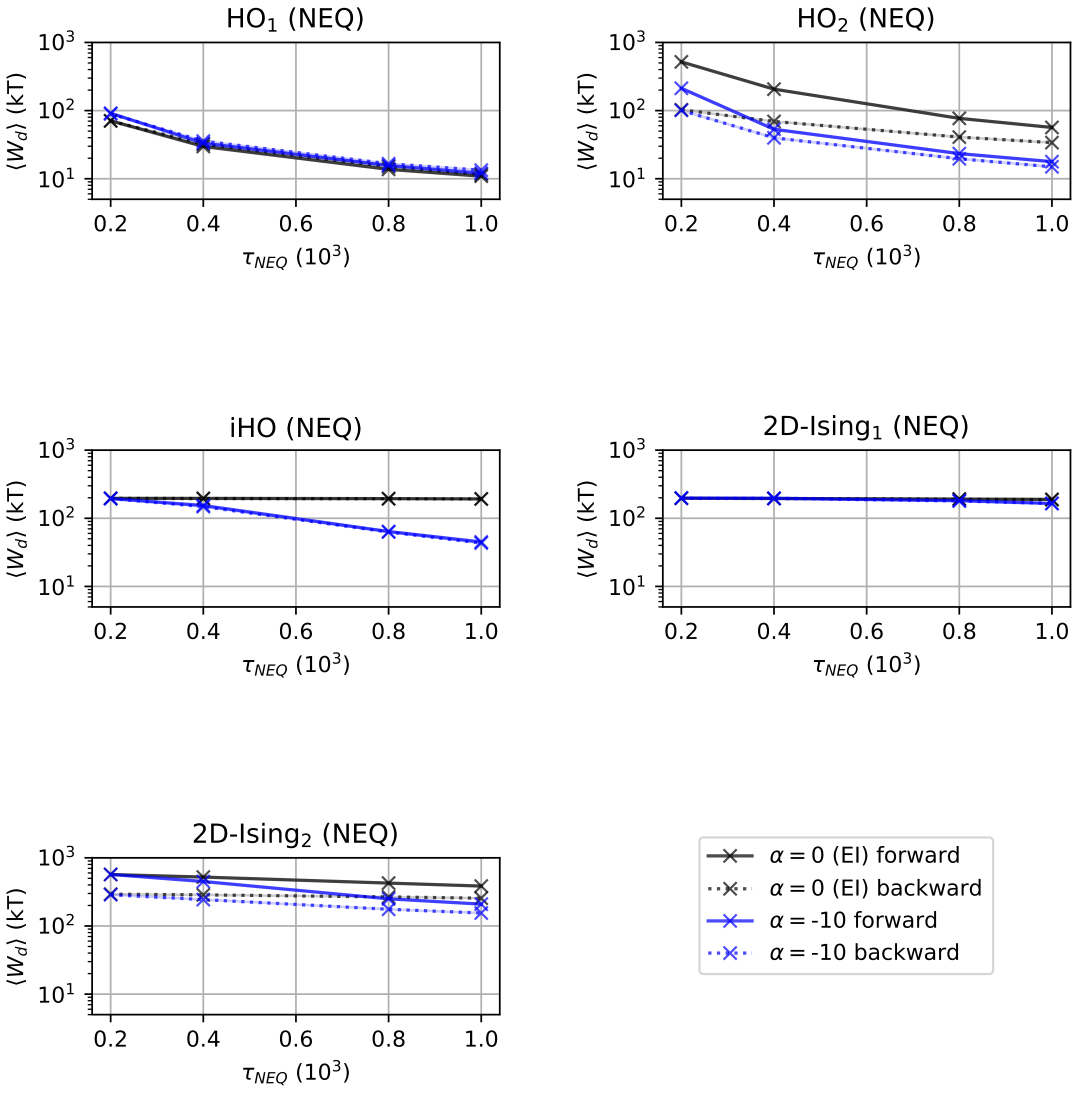

Figure S11: Mean dissipative work in the forward (solid lines) and backward (dashed lines) directions using the EI (black) and EDS (blue) coupling schemes in the five toy systems. Only τ_{NEQ} values between 200 and 1000 are shown for better visualization. The shaded area indicates the 95% confidence interval from bootstrap resampling of the individual forward and backward work values.

S4.4 Estimation Quality is More Sensitive to the Switching Speed than the Number of NEQ Trials

As discussed in Section S4.2, the estimated free-energy differences are less robust when the work distributions do not overlap. In the non-overlapping case, the free-energy difference estimate relies heavily on the few samples with the lowest work values. Although an excessive number of transformations was performed (400 per direction), in all systems, it is important to stress that much fewer transformations are required for simple systems and/or slow transformations.

Figure S12 shows the convergence of the AHFE estimate for a bulky molecule ($mobley\_5456566$) and a small molecule ($mobley\_628086$) at faster (10 ps) and slower (50 ps) switching speeds. At τ_{NEQ} = 10 ps, the bulky molecule shows sudden jumps in ΔG due to the occasional low work transition (Figure S12A) in the sparsely overlapping work distributions (Figure S12C-D). For example, NE-EI sample #366 alone changes the prediction by 10 kJ/mol. In contrast, the estimated ΔG converged for NE-EDS in around 70 samples without abrupt jumps due to the overlapping work distributions (Figure S12D). When the transformation was extended to 50 ps, the convergence of large and small molecules improved significantly compared to 10 ps due to the greater overlap of the work distributions (Figure S12B-H).

In particular, given the same total computational expense $\tau_{total} = N_{NEQ} \cdot \tau_{NEQ}$, increasing τ_{NEQ} appears to be more beneficial than performing more NEQ trials. As the current study only focuses on how the EDS coupling scheme improves the free-energy estimation, we do not discuss the trade-off between these two factors further.

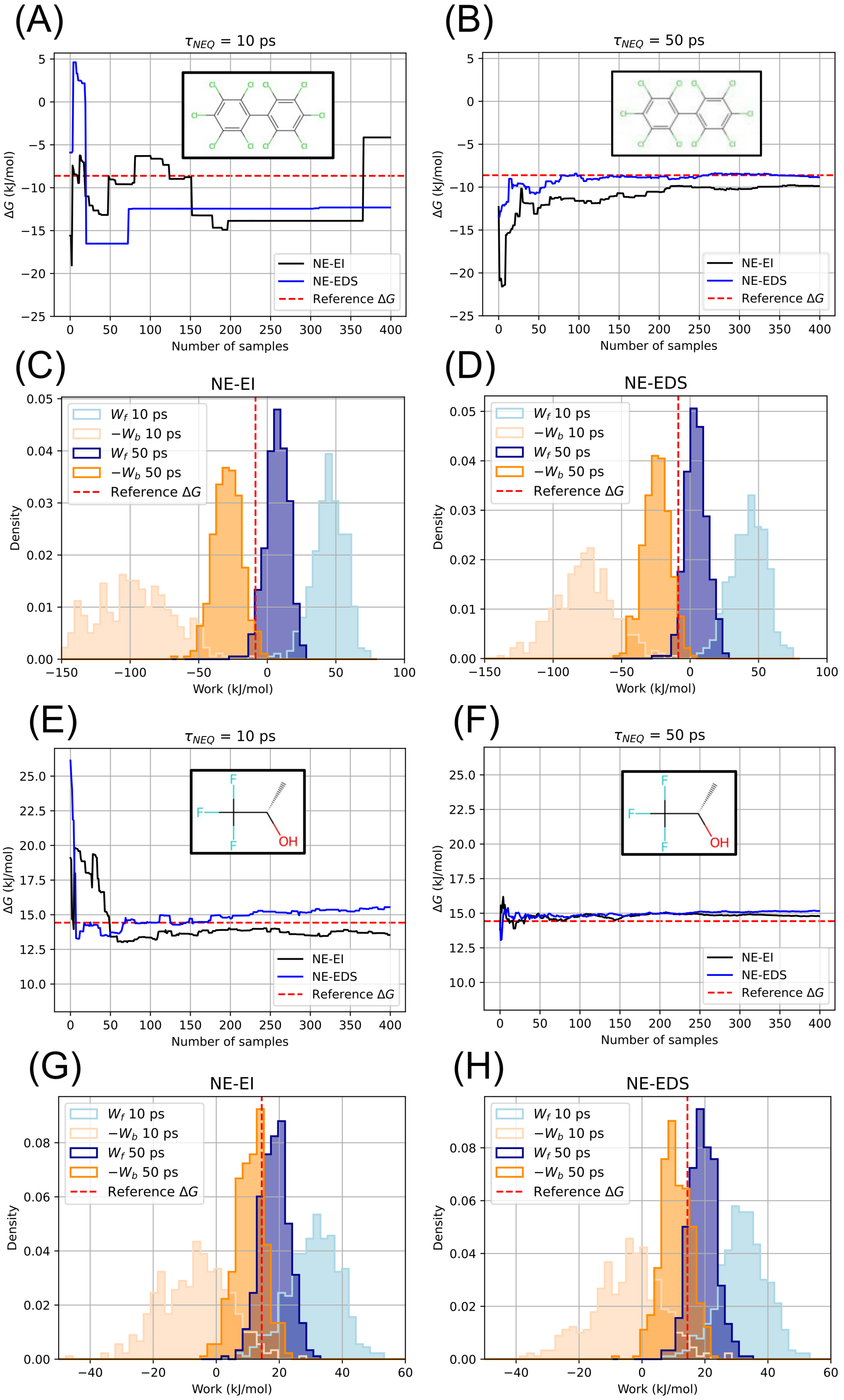

Figure S12: Influence of the transformation speed and number of work samples on the CFT-BAR free-energy difference. (A, B): Convergence profiles of compound $mobley$_5456566 at τ_{NEQ} = 10 ps (A) or 50 ps (B). (C, D): Work distributions of $mobley$_5456566 with NE-EI (C) or NE-EDS (D). (E, F): Convergence profiles of compound $mobley$_628086 at τ_{NEQ} = 10 ps (E) or 50 ps (F). (G, H): Work distributions of $mobley$_628086 with NE-EI (G) or NE-EDS (H). The work distributions in (C, D, G, H) are shown in light colors for the 10 ps transitions and dark colors for the 50 ps transitions.

S5 Detailed Analysis of AHFE Benchmarking

S5.1 Comparison of Reference Computed AHFE Values with Experiment

The reference EQ AHFE estimates (6 ns per λ-point) computed with GAFF 1.8 show overall good agreement with the experimental values (Pearson correlation coefficient r = 0.93, R^2 = 0.81; mean absolute error (MAE) = 5.32 kJ/mol ≈ 1.27 kcal/mol), with a small number of visibly larger deviations, particularly toward the more polar compounds. This level of error is broadly in line with what has been reported for classical fixed-charge force fields [1, 2]. The vertical (computed) error bars are often too small to be visible, suggesting that residual force-field/model errors dominate over statistical uncertainty for these calculations. Note that data for seven of the 642 compounds in the FreeSolv database are missing due to a small number of modeling failures in the automated workflow.

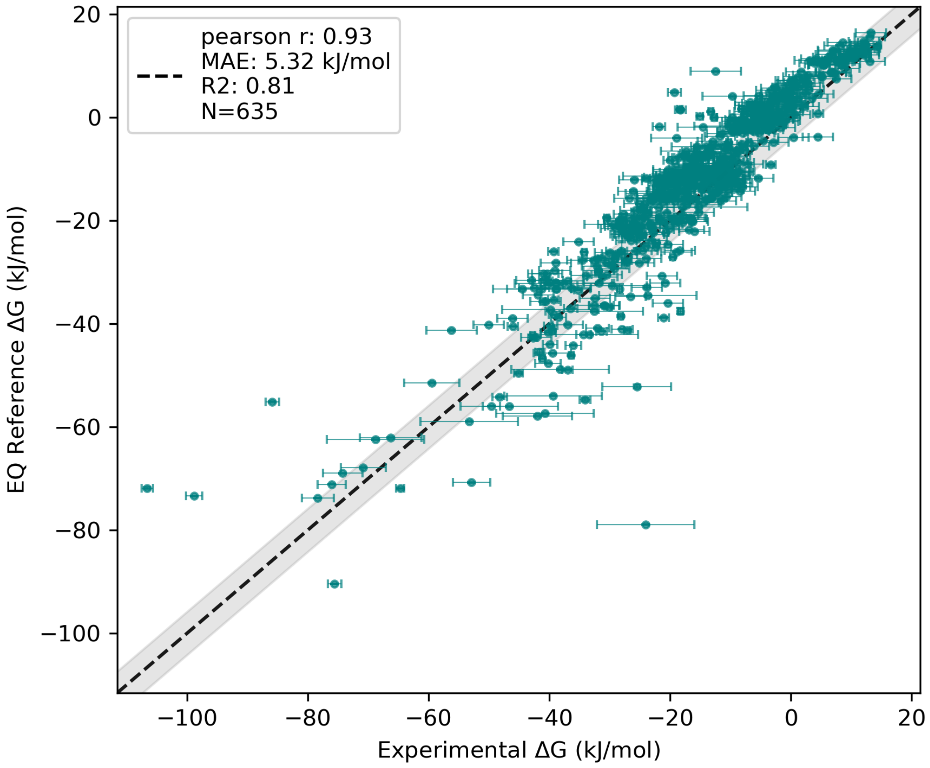

Figure S13: Comparison of the reference EQ AHFE estimates against experimental values for the FreeSolv dataset.

S5.2 AHFE Error Comparison Between Coupling Schemes

For the EQ calculations with six λ-windows, most molecules show similar errors for EI and EDS (Figure S14). A slight tendency toward smaller errors with EDS is visible for many molecules, but this is counterbalanced by a small number of compounds for which EDS performs worse than EI. These outliers persist as the sampling time is increased.

For the NEQ calculations, the improvement is more pronounced (Figure S15). In particular, the largest

NE-EI errors are strongly reduced by NE-EDS, especially at shorter switching times. Thus, the reduced RMSE of NE-EDS is mainly driven by improved behavior for the most difficult compounds, rather than by a uniform small shift across all molecules. At longer switching times, the errors of both methods become smaller and the difference between the two schemes correspondingly decreases.

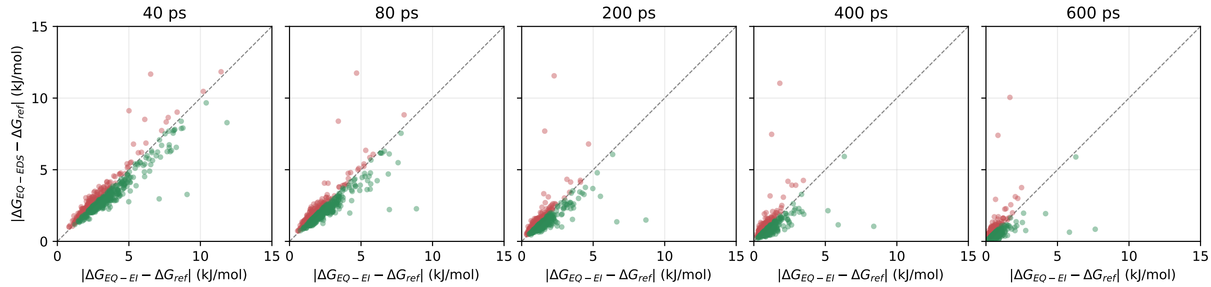

Figure S14: Absolute error in AHFE over subsampled trajectory blocks for EQ-EI and EQ-EDS using six λ-windows and different sampling times per window. Errors are computed relative to the long-EQ MBAR reference values. Each point corresponds to one molecule. Green points indicate cases where EDS has a smaller error than EI, whereas red points indicate cases where EDS has a larger error than EI. The dashed diagonal indicates $y = x$.

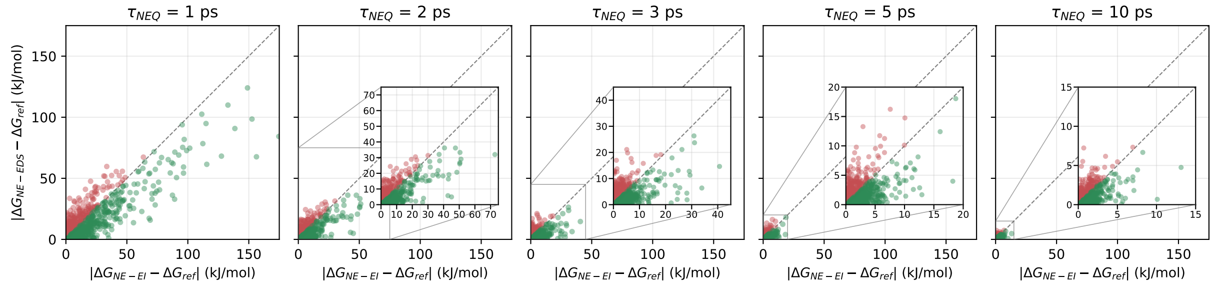

Figure S15: Absolute error in AHFE for NE-EI and NE-EDS at different NEQ switching times. Errors are computed relative to the long-EQ MBAR reference values. Each point corresponds to one molecule. Green points indicate cases where NE-EDS has a smaller error than NE-EI, whereas red points indicate cases where NE-EDS has a larger error than NE-EI. The dashed diagonal indicates $y = x$.

S5.3 Correlation Between Dissipative Work in AHFE NEQ Simulations and 2D Physicochemical Descriptors

The NEQ difficulty of the AHFE switches, as quantified by the mean dissipative work $\langle W_d \rangle$, is primarily determined by molecular size. Polarity descriptors such as topological polar surface area (TPSA) [3] are much less predictive than size-aware descriptors (Figure S16) or simple descriptors such as molecular weight (Figure S17). This supports the mechanistic picture in which the mean dissipation per transformation is dominated by solvent-cavity formation.

This interpretation can be confirmed by decomposing the dissipative work into forward and backward contributions (Figure S18). At $\tau_{\text{NEQ}} = 1$ ps, the forward annihilation process produces substantially less dissipative work than the backward recoupling process, but both remain strongly correlated with χ_v^0. This

indicates that molecular size controls the difficulty of both directions, but that the dominant contribution to the total irreversibility arises from solute insertion. Within this regime, NE-EDS systematically lowers $\langle W_d \rangle$ by adapting the effective coupling when the solvent response lags behind the protocol.

(A)

Descriptor	NE-EI pearson ρ	NE-EDS pearson ρ
chi0v	0.99	0.98
labuteASA	0.98	0.97
MolMR	0.97	0.96
NumValenceElectrons	0.96	0.97
⋮	⋮	⋮
MolWt	0.93	0.92
⋮	⋮	⋮
TPSA	0.40	0.47

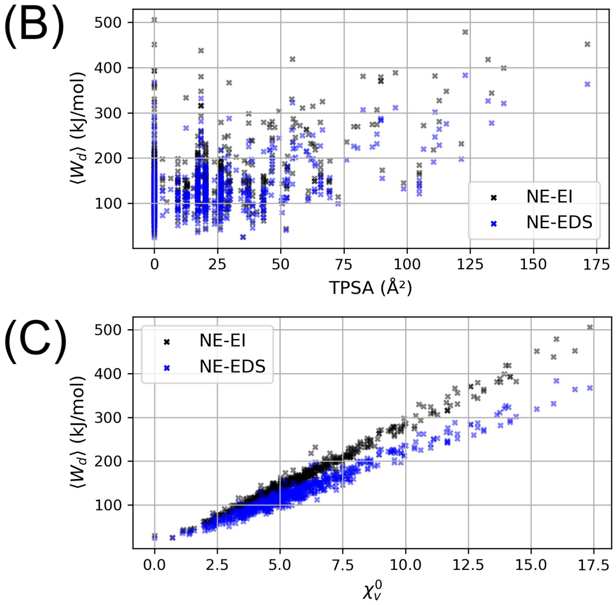

Figure S16: Comparison of mean dissipative work $\langle W_d \rangle$ with 2D physicochemical descriptors. (A): Pearson correlation coefficients between 2D descriptors calculated with the RDKit version 2024.03.6 [4] and $\langle W_d \rangle$ at τ_{NEQ} = 1 ps. (B): Correlation of $\langle W_d \rangle$ with the χ_v^0 descriptor [5], a zero-order descriptor that reflects molecular size by encoding heavy atom types and local connectivities. (C): Correlation of $\langle W_d \rangle$ with TPSA [3], which provides an indication of the polarity of the molecules.

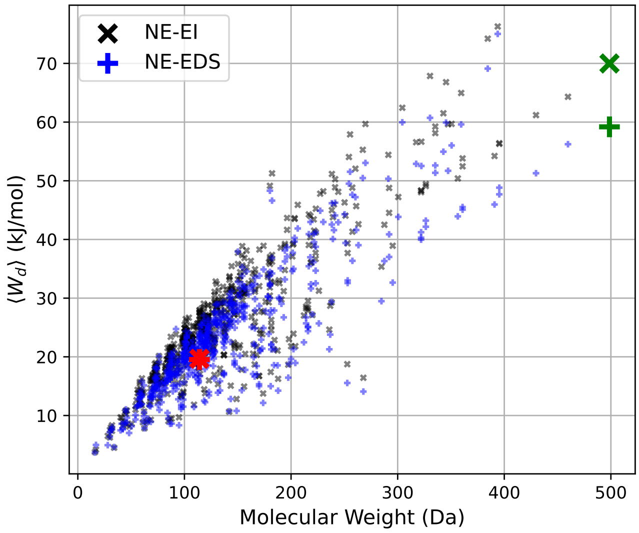

Figure S17: Correlation between the mean dissipative work $\langle W_d \rangle$ at τ_{NEQ} = 10 ps and molecular weight. The data points highlighted in green and red correspond to the AHFE estimates of *mobley_5456566* and *mobley_628086*, respectively, which are used as case studies in this work.

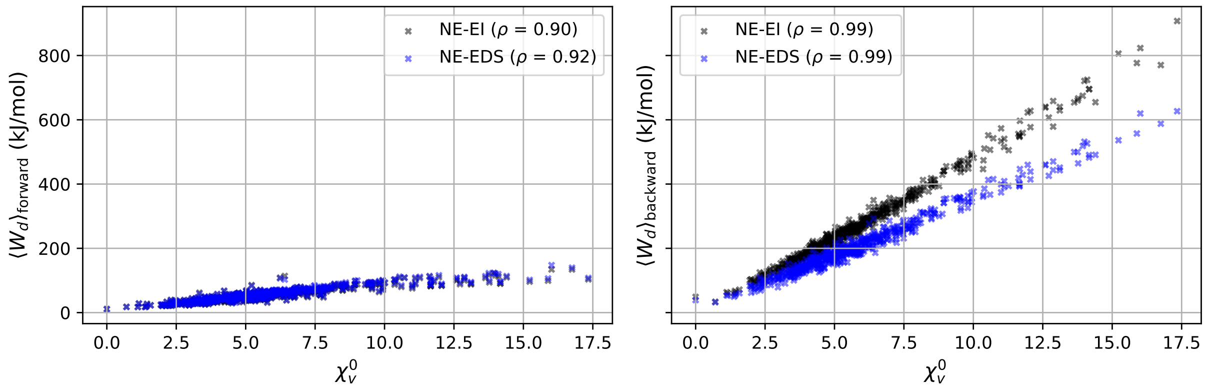

Figure S18: Correlation between directional dissipative work and the valence-weighted connectivity descriptor χ_v^0 at $\tau_{\mathrm{NEQ}} = 1$ ps. The forward direction (left) corresponds to solute annihilation, whereas the backward direction (right) corresponds to solute insertion. Although the forward dissipative work is much smaller in magnitude, both directional contributions remain strongly correlated with molecular size. Pearson correlation coefficients are shown in the legends.

S5.4 Asymmetry of Dissipative Work

The directional decomposition of the dissipative work shows that the irreversibility of the AHFE transformations is strongly asymmetric (Figure S19). For all molecules and switching times, the backward process, corresponding to solute recoupling or insertion, generally produces more dissipative work than

the forward annihilation process. This asymmetry is most pronounced at short switching times and decreases as the protocol time increases.

The reduction in dissipative work obtained with NE-EDS is mainly associated with the backward direction. While the forward dissipation is already comparatively small even at short switching times, NE-EDS systematically lowers the backward dissipative work relative to NE-EI across the FreeSolv dataset. This supports the mechanistic interpretation discussed in the main text: the main source of irreversibility in AHFE is the late and abrupt formation of the solvent cavity during solute recoupling, and the configuration-aware effective coupling in NE-EDS mitigates this bottleneck.

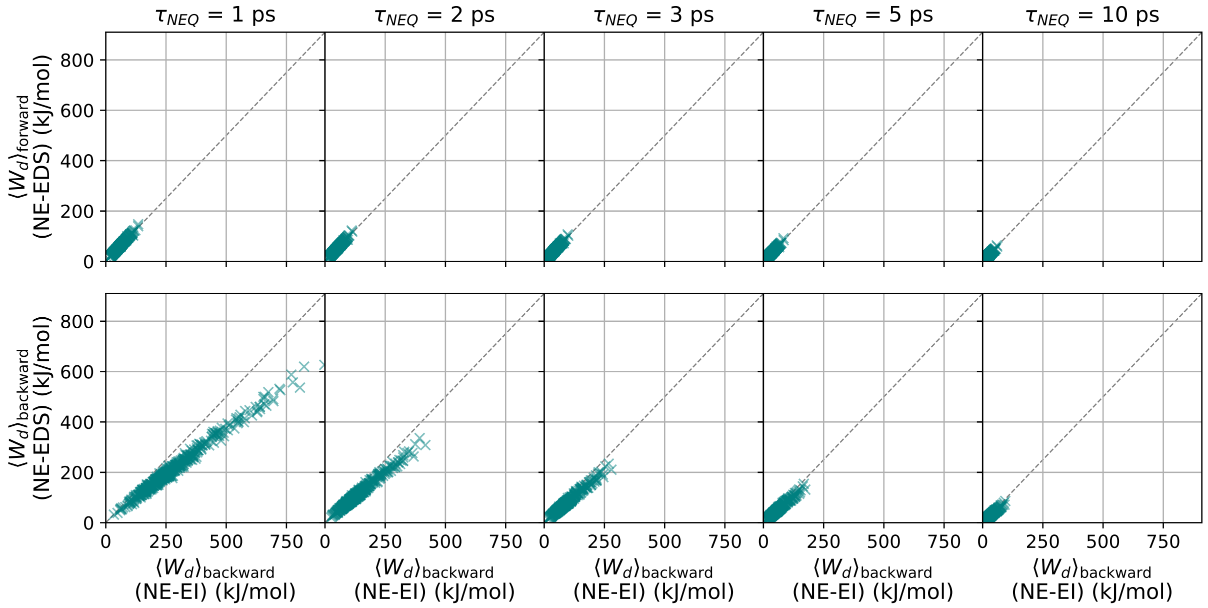

Figure S19: Directional dissipative work in the AHFE NEQ simulations for the FreeSolv dataset. The upper row compares the forward dissipative work from NE-EDS to the backward dissipative work from NE-EI. The lower row compares the backward dissipative work obtained with NE-EDS and NE-EI; points below the diagonal indicate reduced dissipation with NE-EDS.

S6 Sensitivity of NEQ AHFE Results to the Choice of Soft-Core Parameters

All AHFE calculations reported in the main text were performed with the original AMBER18 soft-core formulation with $\alpha = 0.5$ and $\beta = 12$ Å^2. Since the soft-core functional form and its parameters are well known to directly affect the smoothness of the alchemical transformation [6], an additional sensitivity analysis was performed to test whether the observed difference between NE-EI and NE-EDS depends on this choice.

Two alternative soft-core protocols were selected based on the study of Lee et al. [7]. First, the original AMBER18 functional form was retained, but the parameters were changed to $\alpha = 0.2$ and $\beta = 17$ Å^2, as this parameter combination was shown to improve the smoothness of some problematic transformations relative to the AMBER18 default. Second, the AMBER20 smoothstep soft-core formulation was tested with the recommended parameter set $\alpha = 0.2$ and $\beta = 50$ Å^2. This formulation replaces the linear

energy interpolation by a second-order smoothstep function, which was introduced to reduce endpoint and large-gradient artifacts in concerted alchemical transformations of various types.

For each alternative protocol, NEQ AHFE calculations were repeated at $\tau_{\mathrm{NEQ}} = 3$ ps for a randomly selected subset of 50 FreeSolv molecules. Both NE-EI and NE-EDS were evaluated for each molecule, and the resulting free-energy estimates were compared to the same long-EQ MBAR reference values used in the main text.

Figure S20 shows that for this protocol the alternative soft-core choices substantially worsened the accuracy of NE-EI, whereas the accuracy of NE-EDS remained similar across the three tested soft-core settings. The per-molecule errors (Figure S21) show that this difference is mainly caused by a subset of molecules for which NE-EI becomes much less reliable under the alternative soft-core protocols. These results should not be interpreted as a general ranking of the soft-core functional forms, since only one NEQ switching time and a limited subset of molecules were tested. Rather, they indicate that the magnitude of the NE-EDS improvement depends on the smoothness of the underlying alchemical path.

For the present AHFE protocol, the original AMBER18 parameters already appear to provide a comparatively smooth NE-EI pathway, leaving less room for improvement by EDS. When the EI pathway is made less favorable by the alternative soft-core choices tested here, NE-EDS remains comparatively robust and the gap between the two coupling schemes increases. Thus, the magnitude of the EDS advantage is soft-core-dependent, and these investigations support the conclusion that the configuration-aware EDS coupling can reduce the sensitivity of NEQ AHFE estimates to the particular choice of soft-core protocol.

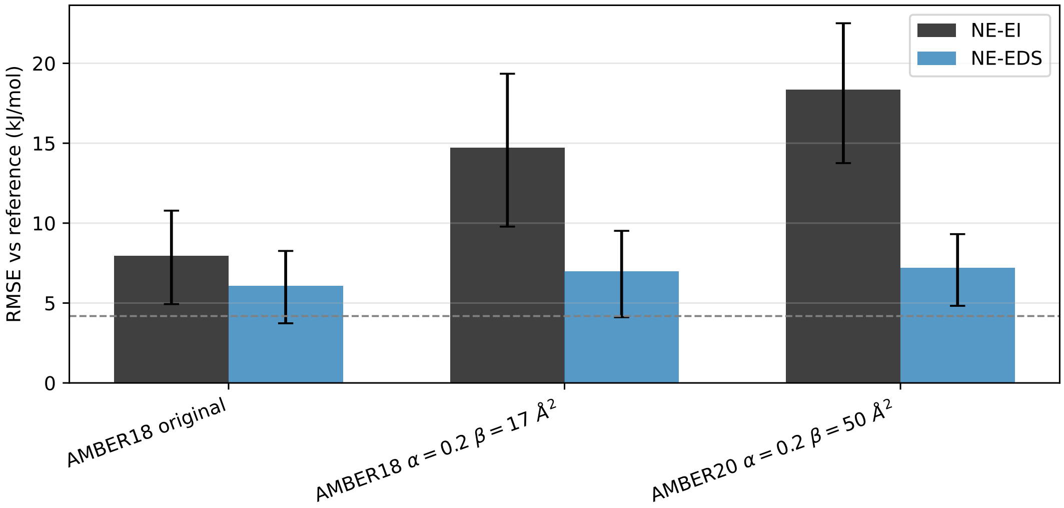

Figure S20: Sensitivity of NEQ AHFE estimates to the soft-core protocol for a randomly selected subset of 50 FreeSolv molecules at $\tau_{\mathrm{NEQ}} = 3$ ps. The RMSE values are computed relative to the long-EQ MBAR reference values for NE-EI and NE-EDS using the original AMBER18 soft-core protocol, an alternative AMBER18 parameter set, and the AMBER20 smoothstep soft-core formulation. Error bars indicate the 95% confidence interval from bootstrap resampling over the 50 molecules. The dashed line indicates chemical accuracy at 4.18 kJ/mol.

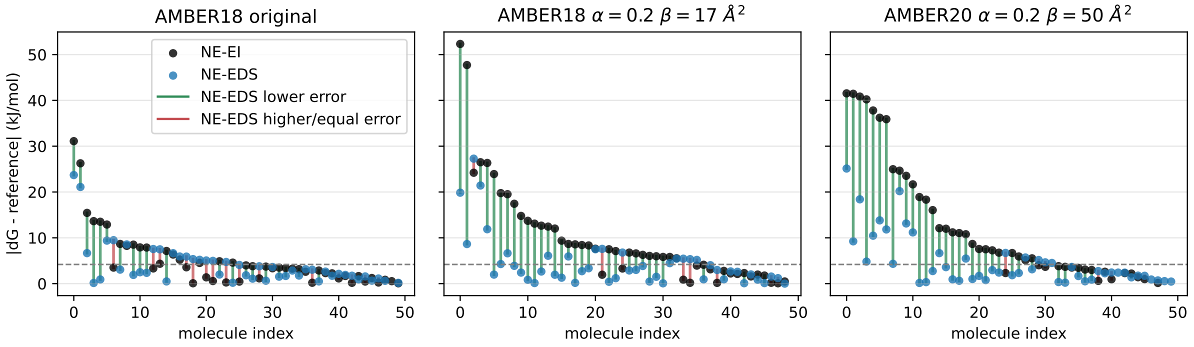

Figure S21: Absolute errors of the individual NEQ AHFE estimates for the three tested soft-core protocols. Within each panel, the 50 molecules are sorted by the largest absolute error observed for either coupling scheme. The dashed line indicates chemical accuracy at 4.18 kJ/mol.

S7 Case Study of the Small Polar Molecule *mobley_628086* in NEQ Simulations

In contrast to the large-molecule example in the main text, the small polar compound *mobley_628086* lies in the low-to-moderate dissipative-work regime (Figure S17), and its NEQ switching shows comparatively mild hysteresis: the forward/backward work distributions overlap more and the ΔG estimate stabilizes rapidly with a narrow confidence interval (Figure S22A-B). Correspondingly, the solvent response is more symmetric between directions (Figure S22E-F), and NE-EDS remains close to the nominal schedule with only modest adaptation (Figure S22G), consistent with the general picture that the largest gains from NE-EDS occur when cavity reorganization becomes rate-limiting.

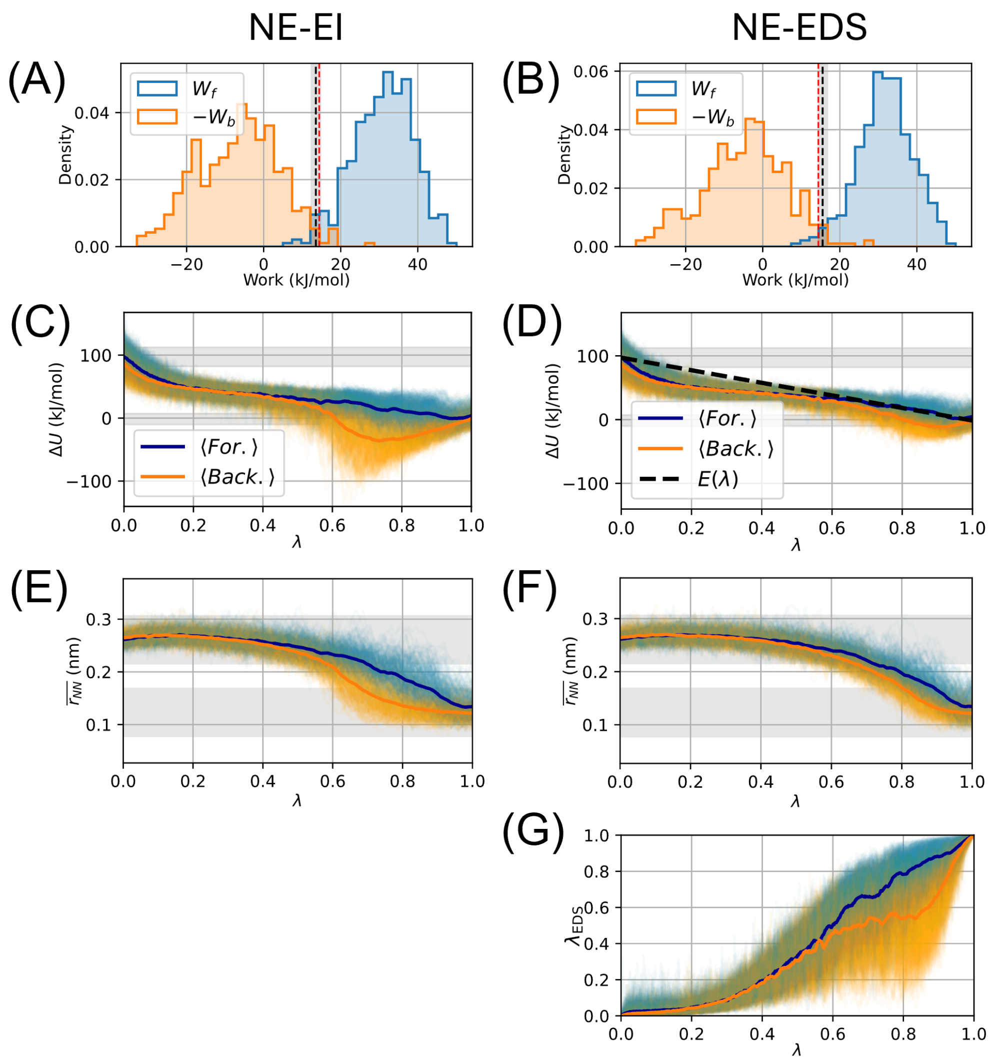

Figure S22: NEQ transformation of a small but polar molecule, *mobley_628086*, at τ_{NEQ} = 10 ps. (A, B): Work distributions from forward (blue) and backward (orange, negative numbers are shown) transformations, reference ΔG (red dashed line), estimated ΔG (black dashed line), and 95% CI (shaded area). (C, D): End-state energy difference and energy offsets (black dashed line). (E, F): Average nearest-neighbor distance between solute atoms and the nearest solvent atoms. (G): Effective λ_{EDS} of the forward and backward trajectories. The ± 1 standard deviation ranges of ΔU and r_{NN} sampled from the end-state simulations are shown with shaded areas (C-F).

S8 Simple Alchemical Transformations

Alchemical protocols that modify solute-solvent interactions generally combine two fundamental operations: scaling electrostatic interactions and scaling van der Waals interactions, with the limiting case of fully decoupling atoms into non-interacting dummies. To better understand the differences we observed in the AHFE calculations, we examined two simplified model systems that isolate these components:

dipole inversion (DI), which probes changes in electrostatics, and *particle insertion (PI)*, which probes the creation of excluded volume in a dense solvent. Together, they provide a minimal set of transformations representative of AHFE calculations for evaluating how EDS compares to EI. We show here the results for a dipole with $q = \pm 1$ e and the insertion of a particle the size of an iodine ion, but similar trends were observed with different dipole charges and particle sizes (data not shown).

S8.1 Dipole Inversion

The first model system is a dipolar molecule composed of two particles with OpenFF 2.2 [8] aliphatic carbon LJ parameters (σ = 0.338 nm, ϵ = 0.455 kJ/mol) and charges of ± 1 e dissolved in a water box. The alchemical transformation takes place by linearly changing the charge from +1 and –1 (end-state A) to –1 and +1 (end-state B). The positions of the two particles were kept constrained to their starting coordinates aligned on the z-axis with a separation of 0.2 nm, while the solvent rearranges its configuration to adapt to the change in dipole moment. Both end states were initially sampled for 100 ps, after which 400 NEQ simulations per direction were performed for various τ_{NEQ} values (1, 2, 3, 5 or 10 ps). At each frame, the electrostatic field vector was computed by placing a non-interacting charged probe in the middle of the two particles, whose z-component of the force ($E_z(\mathbf{r})$) was used as a progress coordinate to measure the solvent response to a change in the solute dipole moment.

Figure S23 shows the resulting trajectories both in the energy domain and through the electrostatic field progress coordinate. In both NE-EI and NE-EDS, $E_z(\mathbf{r})$ and $\Delta U(\mathbf{r})$ vary almost linearly along the switching coordinate. For NE-EI, hysteresis is uniform along the trajectory (both $E_z(\mathbf{r})$ and $\Delta U(\mathbf{r})$ differ on average by an approximately constant amount between the forward and reverse directions), whereas NE-EDS slightly reduces this hysteresis, and this reduction is most pronounced around the midpoint of the transformation. However, this small difference does not lead to a significant reduction in the amount of irreversible work produced nor to a significant improvement of the estimated free-energy difference (Figure S25).

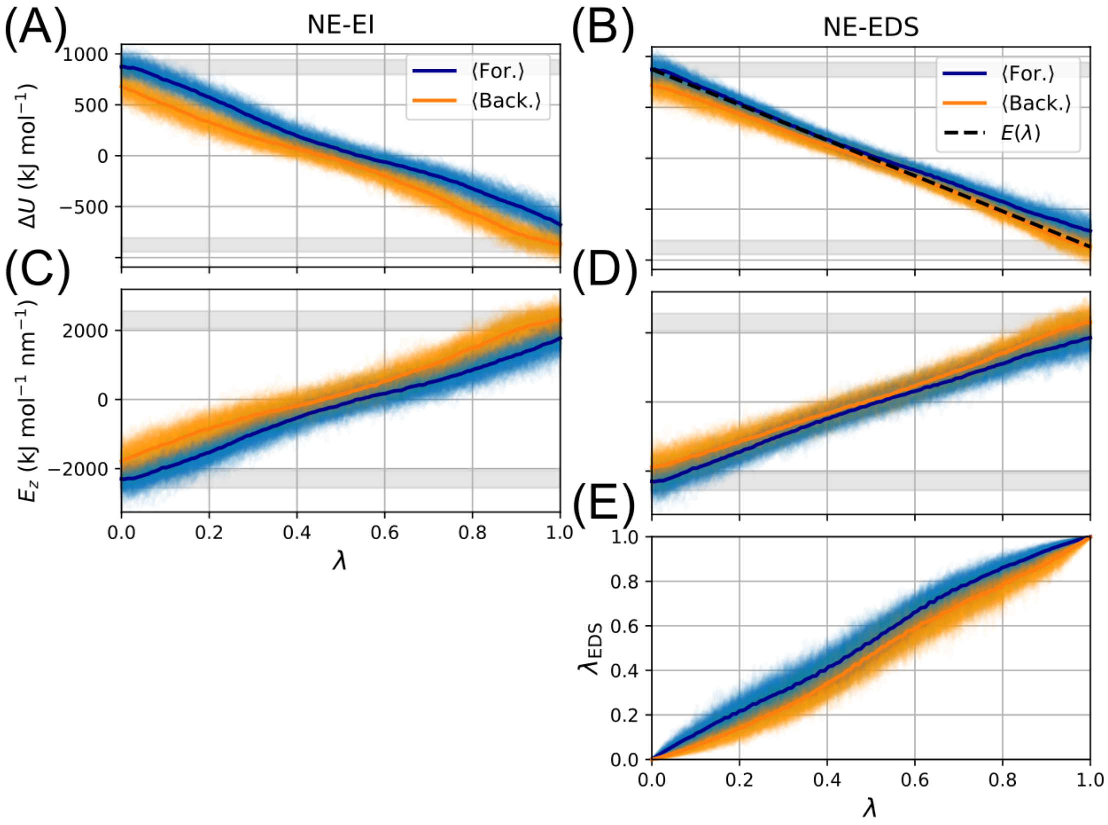

Figure S23: 400 NEQ trajectories (blue: forward, orange: backward) of the dipole inversion process for τ_{NEQ} = 2 ps. (A, B): End-state energy difference, with the linearly interpolated energy offsets as a black dashed line. (C, D): Electric field of the EI (C) and EDS (D) coupling schemes. (E): Effective λ_{EDS} of the forward and backward trajectories. The ± 1 standard deviation ranges of ΔU and Ez sampled from the end-state simulations are shown with shaded areas (A-D).

S8.2 Particle Insertion

Inserting a LJ particle into a condensed phase represents a process for which the choice of alchemical pathway is much more important than for DI. If the interactions are turned on by naively interpolating a hard-core LJ potential, the potential-energy landscape develops singularities as the particle "appears" within the excluded volume of solvent molecules, causing numerical instabilities and extremely large forces, which translate into very large irreversible work production in NEQ. This so-called van der Waals end-point problem has been extensively documented [9, 10]. To overcome it, soft-core potentials are typically employed to modify the short-range repulsion so that interaction energies remain finite even when atoms coincide, producing smoother free-energy profiles and greatly improved numerical stability, as well as more reversible transitions in NEQ. Although these modified potentials provide a more efficient alchemical pathway compared to linear scaling, they are typically implemented as a modification of the end-state LJ potentials coupled with standard EI. In the following, we show that soft-core potentials can further benefit from a smoother coupling scheme, namely EDS.

A neutral atom with LJ parameters of an I^- ion in OpenFF 2.2 [8] was transformed from the fully coupled state (λ = 0) to a fully decoupled state (λ = 1) in a water box by scaling down its intermolecular interactions with solvent molecules. As for DI, the end-states were sampled for 100 ps, and 400 NEQ trajectories were initiated from the obtained samples in each direction. As with the AHFE protocol, the EDS potential was defined with only a single non-zero state, $U_A(\mathbf{r}, \lambda)$ = $U_{LJ}(\mathbf{r}, \lambda)$ and U_B = 0. At λ = 1, U_{EDS} = U_B = 0; therefore, the solute is fully decoupled from the solvent (solvent-solvent and solute-solute interactions, however, are still unperturbed). For intermediate states, soft-core interactions were used with a soft-core α-value of 0.2 to mimic a hard insertion (see the Methods section in the main text). To represent the change in configurational space required upon insertion / annihilation of a particle, the distance to the nearest neighbor (r_{NN}) of the solute atom was measured at every frame of the simulation.

The resulting trajectories at τ_{NEQ} = 2 ps (Figure S24) exhibit a significant hysteresis between the forward and reverse directions. In the forward process (annihilation), the trajectories appear uniformly out-of-equilibrium as the solvent cavity remains consistently larger than in equilibrium at all λ-values. On the contrary, for most paths in the reverse process (insertion), the solvent initially fails to open a cavity at all, keeping the solute overlapped with water until the cavity abruptly forms much later along the λ-coordinate. This results in highly dissipative transformations where irreversible work is produced by forcing overlapping particles to interact before the solvent has enough time to relax its configuration. The NE-EDS coupling helps prevent these extreme cases by modulating the effective λ based on the difference between the instantaneous energy difference $\Delta U(\mathbf{r}, \lambda) = U_B(\mathbf{r}, \lambda) - U_A(\mathbf{r}, \lambda) = -U_{LJ}(\mathbf{r}, \lambda)$ and the energy offset $E(\lambda)$. This results in a considerably reduced amount of irreversible work produced for these transformations, since W_d is consistently 25-50% higher for NE-EI than for NE-EDS across the investigated τ_{NEQ} values (Figure S25).

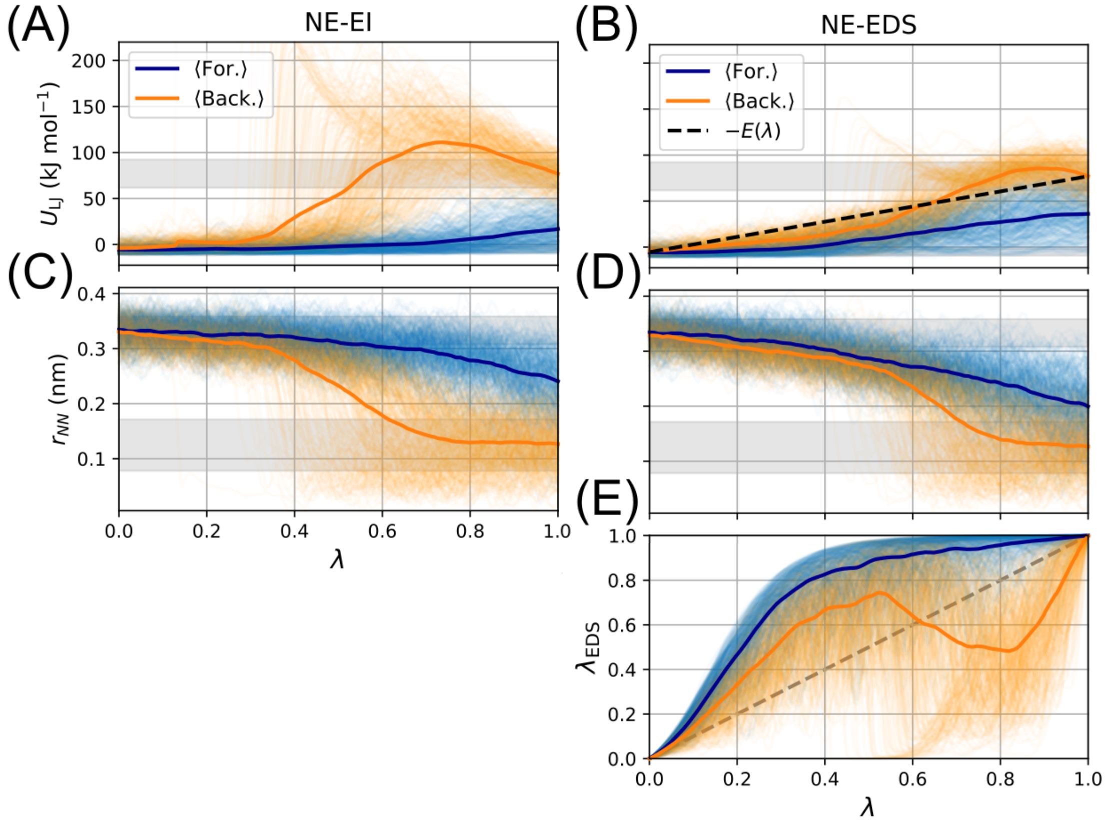

Figure S24: 400 NEQ trajectories (blue: forward, orange: backward) of the particle insertion process for τ_{NEQ} = 2 ps. (A, B): End-state potential energy, with the linearly interpolated energy offsets as a black dashed line. (C, D): Distance between the solute particle and the nearest solvent atom in the EI (C) and EDS (D) coupling schemes. (E): Effective λ_{EDS} of the forward and backward trajectories. The ± 1 standard deviation ranges of ΔU and r_{NN} sampled from the end-state simulations are shown with shaded areas (A-D).

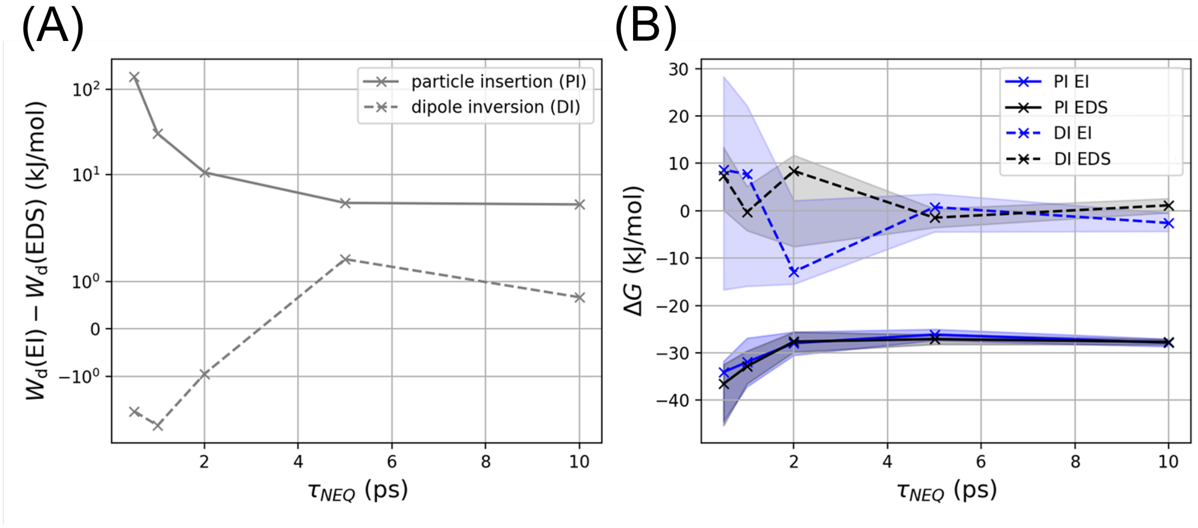

Figure S25: Dissipative work (A) and free-energy estimates (B) of the dipole inversion (DI) and particle insertion (PI) systems.

References

[1] Duarte Ramos Matos, G.; Kyu, D. Y.; Loeffler, H. H.; Chodera, J. D.; Shirts, M. R.; Mobley, D. L. Approaches for Calculating Solvation Free Energies and Enthalpies Demonstrated with an Update of the FreeSolv Database. *J. Chem. Eng. Data* **2017**, *62*, 1559–1569.

[2] He, X.; Man, V. H.; Yang, W.; Lee, T.-S.; Wang, J. ABCG2: A Milestone Charge Model for Accurate Solvation Free Energy Calculation. *J. Chem. Theory Comput.* **2025**, *21*, 3032–3043.

[3] Ertl, P.; Rohde, B.; Selzer, P. Fast Calculation of Molecular Polar Surface Area as a Sum of Fragment-Based Contributions and Its Application to the Prediction of Drug Transport Properties. *J. Med. Chem.* **2000**, *43*, 3714–3717.

[4] Landrum, G. et al. Rdkit/Rdkit: 2024_03_6 (Q1 2024) Release. Zenodo, 2024.

[5] Hall, L. H.; Kier, L. B. The Molecular Connectivity Chi Indexes and Kappa Shape Indexes in Structure-Property Modeling. In *Reviews in Computational Chemistry*. John Wiley & Sons, Ltd 1991; pp 367–422.

[6] Lundborg, M.; Lidmar, J.; Hess, B. On the Path to Optimal Alchemistry. *Protein J.* **2023**, *42*, 477–489.

[7] Lee, T.-S.; Lin, Z.; Allen, B. K.; Lin, C.; Radak, B. K.; Tao, Y.; Tsai, H.-C.; Sherman, W.; York, D. M. Improved Alchemical Free Energy Calculations with Optimized Smoothstep Softcore Potentials. *J. Chem. Theory Comput.* **2020**, *16*, 5512–5525.

[8] McIsaac, A. R.; Behara, P. K.; Gokey, T.; Cavender, C.; Horton, J.; Wang, L.; Westbrook, B. R.; Thompson, M. W.; Osato, M.; Baumann, H. M.; Jang, H.; Wagner, J.; Cole, D.; Bayly, C.; Mobley, D. Openforcefield/Openff-Forcefields. 2024.

[9] Straatsma, T. P.; Berendsen, H. J. C.; Postma, J. P. M. Free Energy of Hydrophobic Hydration: A Molecular Dynamics Study of Noble Gases in Water. *J. Chem. Phys.* **1986**, *85*, 6720–6727.

[10] Beutler, T. C.; Mark, A. E.; van Schaik, R. C.; Gerber, P. R.; van Gunsteren, W. F. Avoiding Singularities and Numerical Instabilities in Free Energy Calculations Based on Molecular Simulations. *Chem. Phys. Lett.* **1994**, *222*, 529–539.
